# Dissociation Energies via Embedding Techniques

**DOI:** 10.1021/acs.jpca.4c02851

**Published:** 2024-10-15

**Authors:** Florian Feyersinger, Peter E. Hartmann, Johannes Hoja, Peter Reinholdt, Florian Libisch, Jacob Kongsted, Peter Puschnig, A. Daniel Boese

**Affiliations:** † Department of Chemistry, University of Graz, Heinrichstraße 28/IV, 8010 Graz, Austria; ‡ Department of Physics, University of Graz, 8010 Graz, Austria; § Department of Physics, Chemistry and Pharmacy, University of Southern Denmark, Campusvej 55, 5230 Odense M, Denmark; ∥ Institute for Theoretical Physics, TU Wien, 1040 Vienna, Austria

## Abstract

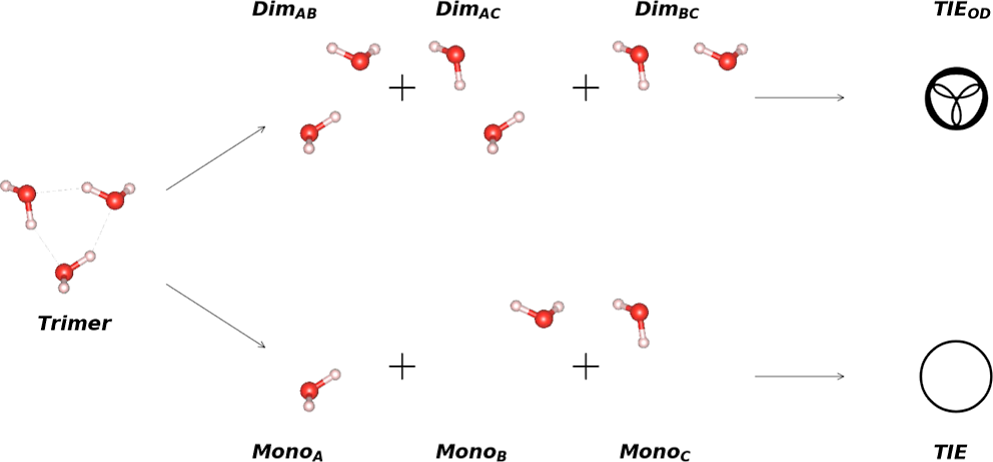

Due to the large number of interactions, evaluating interaction energies for large or periodic systems results in time-consuming calculations. Prime examples are liquids, adsorbates, and molecular crystals. Thus, there is a high demand for a cheap but still accurate method to determine interaction energies and gradients. One approach to counteract the computational cost is to fragment a large cluster into smaller subsystems, sometimes called many-body expansion, with the fragments being molecules or parts thereof. These subsystems can then be embedded into larger entities, representing the bigger system. In this work, we test several subsystem approaches and explore their limits and behaviors, determined by calculations of trimer interaction energies. The methods presented here encompass mechanical embedding, point charges, polarizable embedding, polarizable density embedding, and density embedding. We evaluate nonembedded fragmentation, QM/MM (quantum mechanics/molecular mechanics), and QM/QM (quantum mechanics/quantum mechanics) embedding theories. Finally, we make use of symmetry-adapted perturbation theory utilizing density functional theory for the monomers to interpret the results. Depending on the strength of the interaction, different embedding methods and schemes prove favorable to accurately describe a system. The embedding approaches presented here are able to decrease the interaction energy errors with respect to full system calculations by a factor of up to 20 in comparison to simple/unembedded approaches, leading to errors below 0.1 kJ/mol.

## 1 Introduction

The description of noncovalent interactions and larger systems has become ever more important in the subjects of modern natural sciences. Fields such as biosciences,^1,2^ nanosciences,^3^ interfaces,^3−5^ as well as any computation of liquids^6^ heavily rely on molecule–molecule interactions. This is especially true due to the sheer number of molecules present in these systems. Thus, it comes as no surprise that the accurate representation of noncovalent interactions nowadays has become one of the major concerns of computational and quantum chemistry.

Small errors in interaction energies (of molecular dimers, trimers, tetramers, and up to multimers) may sum up and hence lead to a considerable offset. This implies that there is a high demand for a cheap but at the same time still accurate method to determine interaction energies and gradients. The desired accuracy could, for example, be reached by density functional theory (DFT) utilizing hybrid functionals or post-Hartree–Fock methods, but these approaches are often too time-consuming to apply to large isolated clusters or periodic systems. One solution is to partition a large system into smaller subsystems, with an appropriate formalism to describe subsystem interactions. This has led to an increasing number of approaches and applications in recent years.^7−21^

The unfavorable scaling of more accurate electronic structure methods with system size often precludes the application of highly accurate approaches to an entire system of interest. To reach a suitable compromise between accuracy in important areas and speed in less important regions, one partitions a system into manageable parts, with the interaction between parts (or subsystems) described with a less accurate yet also numerically less demanding method. Several different schemes have been proposed to describe the interaction between subsystems,^7^ with several different naming conventions throughout the literature. One can, for example, distinguish between so-called subsystem approaches that partition a system into equal parts that are treated with a more advanced technique than their interaction^22,23^ and embedding methods that typically focus on a cluster of interest, for example, an adsorption site in catalysis (treated with a high-level method) embedded into a surrounding environment.^7,24^

For the present discussion, we partition the molecular cluster into subsystems composed of one or more monomers (i.e., we do not split up the molecules). As we compare many different procedures, we use the term “embedding” quite generally as some approach to model the interaction between the subsystems. We are able to significantly speed up hybrid DFT calculations and still obtain highly accurate results for molecular crystal structures and energies by using multimer embedding methods.^25,26^

The smallest noncovalently bound system is thus a dimer, where only the interaction of a single monomer with another monomer is present. The interaction energy *E*^inter^ within a cluster of *N*_mon_ monomers is, at first, often approximated as the sum of all pairwise interaction energies *E*_*ij*_^inter^ between monomers *i* and *j*
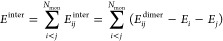
1where *E*_*ij*_^dimer^ is the total energy of the dimer composed of monomers *i* and *j*, while *E*_*i*_ and *E*_*j*_ are the respective monomer energies. Please note that this way, possible double-counting of the monomers is removed. This is only exact if either trimer and higher interactions are zero or if the cluster is a dimer.

The next larger system of interest would be a trimer, in which interaction energy terms, such as induction, dispersion, or exchange repulsion between monomers, are nonadditive beyond the dimer interaction. This nonadditive behavior is neglected in eq 1; therefore, additional interaction terms need to be considered. Correctly describing trimer or, in general, multimer interaction energies is key to obtaining meaningful values from embedding methods. Thus, from the point of view of studying the fundamentals of the increasingly popular many-body expansion methods,^27−29^ trimers represent the lowest-order corrections to consider beyond dimers, providing insight toward a *systematical* approach to embedding.

In this work, twenty-seven geometries of trimers from the 3B-69 benchmark set^30^ and an extra water as well as ammonia trimer were investigated to obtain data that can be considered in a statistical analysis of obtained interaction energies. For the evaluation of different embedding methods (e.g., like electrostatic embedding) and schemes (and thus utilized equations) for these methods, we restrict ourselves to energies obtained by DFT since it is the most applicable method to a high number of molecules in (periodic) systems of interest.^31−34^ We will utilize a generalized gradient approximation (GGA) functional for our “low level of theory” and a hybrid functional for representing the “high level of theory”. We aim to demonstrate how embedding allows avoiding an expensive hybrid calculation of the entire system and show that simple embedding methods are already able to accurately reproduce the benchmark energy of the full hybrid calculation. Deviations come from errors in describing the interaction energy among the subsystems. More involved embedding schemes and methods allow for drastically decreasing this interaction energy error to a predefined “true” value, in combination with the right scheme, by more than 1 order of magnitude.

## 2 Embedding Methods

First, we give an overview of different embedding methods as well as the applied schemes to approximate the interaction energy. The term “schemes” in this paper corresponds to a number of equations to approximate (trimer) interaction energies. These are usually utilized to correct for double-counting effects. All interaction energy approximations, i.e., schemes, have a corresponding schematic illustration given in the Supporting Information, Section S2, for better understanding. We denote in the discussion of the following schemes all total energies with the letter *E* and interaction energies with Ξ. For monomers and dimers, indices *i* and *j* denote the noninteracting subsystems involved; for example, *E*_*i*_ refers to the total energy of the monomer *i*, while Ξ^dimer^ refers to the interaction energy of the dimer composed of the monomers *i* and *j*. The most simple scheme can then be written as
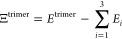
2Reference values for the trimer interaction energy Ξ^trimer^ are obtained from eq 2 by subtracting from the total energy of the trimer *E*^trimer^ all monomer energies *E*_*i*_. This definition of the trimer interaction energy in eq 2 also includes two-body interactions and therefore represents all interactions in a trimer.

We use throughout the article “exact two-body” and “exact three-body” interactions to refer to energy contributions obtained exclusively at the DFT level without explicitly adding long-range dispersion effects via *a posteriori* dispersion models since we want to focus on embedding using only pure density functionals. We provide a variety of different schemes to calculate the trimer interaction energy in embedding approaches. Due to the large number of possibilities, we limit ourselves in the main text to simple schemes (formulas), while the results for additional schemes can be found in the Supporting Information in Section S2. Furthermore, we have used several groups of embedding methods, going from purely mechanical embedding to projection-based embedding and (projection-based) density embedding. These different embedding types are summarized below.

### 2.1 Mechanical Embedding

We define mechanical embedding as a fragmentation without interaction between subsystems: a dimer is split into two separate monomers and a trimer into either three monomers or three dimers.

A simple approximation Ξ_d_ for the trimer interaction energy Ξ^trimer^ can then be obtained from the three dimers with their corresponding pairwise interaction energy *E*_*ij*_^dimers^ according to
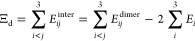
3This corresponds to eq 1 on the level of the trimer interaction energy composed of the dimers, where we include the respective interaction energies of the three dimers from their constituent monomers. This scheme includes all two-body interactions on the embedded level but does not account for any three-body interactions. All total energies of the monomers *E*_*i*_ are overcounted, i.e., when adding the total energies of three dimers *E*_*ij*_^dimers^, each monomer appears twice. Consequently, to obtain only the trimer interaction energy, we have to subtract the monomer energies twice in the formula. For example, for a trimer, the dimers 1 and 2, 2 and 3, and 1 and 3 are present, and each monomer is present and counted twice. A similar formula on the monomer level would leave us with no interaction energy whatsoever; hence, Ξ_m_ does not exist when investigating interaction energies.

It is also possible to use an ONIOM (“our own N-layered integrated molecular orbital + molecular mechanics”)-like^35,36^ approach, in which the full system (“trimer”) is calculated at a low level of theory (superscript low) and all subsystems (like dimers “dimers”) at a higher level (superscript high). Such methods are also called “subtractive schemes”, which contrast the “additive schemes”, in which the interaction energies are merely added up. Thus, the difference between high and low levels for the subsystems corrects for the low-level interaction of the complete system. This usually results in a reasonably good approximation for the high-level results of the complete system and can reduce the computational cost.

In other words, for this scheme, the dimer interactions are calculated at the high level with eq 3, whereas they are computed at the low level of theory exactly via eq 2, arriving at Ξ_low_^trimer^. The ONIOM approach/subtractive scheme (ONIOM-dimer, abbreviated by Ξ_od_) for the simple fragmented method is given by
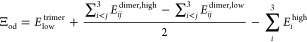
4(see the Supporting Information for a graphical representation). This method has already been successfully applied to molecular crystals.^25,26,37,38^ In a similar manner, just monomer energies can be embedded, arriving at the ONIOM-monomer (om) level Ξ_om_. Here, the trimer interaction energy stems mainly from the low-level method
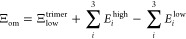
5For the sake of clarity and brevity, we describe here only the basic schemes for each type of embedding. We provide a full listing including all descriptions in Section S2 of the Supporting Information.

### 2.2 Electrostatic and Polarizable Embedding

In order to go beyond the purely mechanical approach presented above, direct interactions between subsystems can be split into different contributions that can then be individually approximated via embedding. We distinguish different contributions from intermolecular interactions since this describes our approach of further splitting the here presented embedding methods into groups quite well. Specifically, the interaction energy is composed of the following contributions
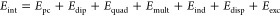
6with the following meaning: electrostatics from point charges (*E*_pc_), dipoles (*E*_dip_), quadrupoles (*E*_quad_), and higher order multipoles (*E*_mult_), as well as induction (*E*_ind_), dispersion (*E*_disp_), and exchange (*E*_exc_), respectively. Note that the exchange term is purely nonclassical and emerges from overlapping orbitals, while we note that long-range dispersion cannot adequately be described by common density functionals and typically requires the usage of an additional dispersion model.

As mentioned above, trimers can be split into three different dimers, resulting in exact two-body interaction, which should, given our definition of eq 2, yield results reasonably close to reference values. We expect to see a decrease in error with respect to the reference value by increasing the number of interaction terms of eq 6 included in the embedding approach.

Taking a look at the electrostatic part, the embedding method corresponding to point charges will be called point charge embedding (“PCE”), dipoles will lead to dipole charge embedding (“DCE”), and quadrupoles will therefore result in quadrupole charge embedding (“QCE”). Leading to a common embedding approach, electrostatic embedding, which is the usage of point charges at atom or even molecule positions for the monomers which are not included (monomers in other subsystems) to evaluate *E*_pc_ from eq 6. This allows the subsystems to electrostatically interact with each other, whereas other contributions are still neglected. The point charges can be obtained from, for example, Mulliken population analysis. When using other charges, such as natural bond order charges, we find a similar accuracy. Point charges yield not only an electrostatic approximation for two-body interaction in the case of a dimer but also the electrostatic influence on the two-body, as well as an approximation of the three-body interaction in the case of a trimer.

Here, we apply this method by utilizing both the TURBOMOLE and the DALTON codes, where the point charges are located at atomic positions. Since we have several possibilities of splitting our system, we also have some extra schemes to consider, as described and illustrated in the Supporting Information. The energies of each subsystem do not include the self-energy of the used point charges. Thus, in the additional schemes, we can subtract subsystem energies, arriving at relations more complicated than the rather simple eq 2. Because of these double-counting effects, Ξ_d_, Ξ_od_, and Ξ_om_ of eqs 3, 4, and 5, respectively, have to be changed accordingly when including embedding. This is true not only for electrostatics but also for other embedding methods. When doing so, we arrive at Ξ_pdim_, Ξ_odp_, and Ξ_omp_. Ξ_pdim_, for example, is adding all embedded dimers *E*_*ij*,embedded_^dimer^ and then subtracting each of the monomers *E*_*i*_, such as
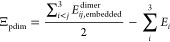
7Ξ_omp_, in contrast, is adding the difference of the embedded high-level and low-level monomer contributions to the low-level trimer energy in an ONIOM scheme
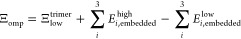
8Finally, Ξ_odp_ adds the difference of the embedded high-level and low-level dimer contributions to the low-level trimer energy in an ONIOM scheme
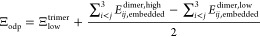
9

We also find good results for the “pd3” additive scheme, in which a new trimer interaction energy Ξ_pd3_ is obtained
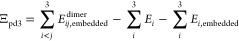
10Here, the embedded dimer interaction energies (denoted by the subscript) are added up, and the double counting via embedded monomers is subtracted. For the subtractive schemes, a similar equation can be constructed, obtaining Ξ_odww_
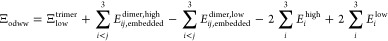
11which combines the low-level trimer interaction Ξ_low_^trimer^ with the difference in low- (*E*_*ij*,embedded_^dimer,low^) and high-level embedded dimer-interaction energy (*E*_*ij*,embedded_^dimer,high^) and again subtracts the difference between the monomers, which were double-counted (*E*_*i*_^high^ and *E*_*i*_^low^). For more details on these (and more complicated) schemes, refer to the respective Figure schematics and derivations in the Supporting Information.

Further electrostatic embedding includes a multipole expansion appending terms of higher order to the electrostatic potential. An expansion of the previous method introduces, therefore, a dipole term, *E*_dip_, in eq 6, leading to dipole embedding. The calculation schemes for this and the following methods are identical to the ones used for point charge embedding.

One can further expand the multipole approach from dipoles to quadrupoles, including the *E*_quad_ value of eq 6. Although higher-order multipole expansions are in principle available, we will stop applying the electrostatic interaction of the subsystems at this level.

To go beyond pure electrostatic embedding, the polarization/induction of the outer region and its effect on the core region can be introduced in addition to the description of the permanent electrostatics based on multipole descriptions. This is achieved by applying anisotropic dipole–dipole polarizabilities in the effective operator.^39,40^ This embedding will be called polarizable embedding (“PE”).

### 2.3 Density and Polarizable Density Embedding

A well-known difficulty when applying electrostatic embedding is the electron spill-out^41−43^ problem caused by the exchange terms, which is counteracted in the density embedding (“DE”) method by adding repulsion contributions from the environment regions. Previously introduced static multipoles are replaced by their charge densities, and the self-energy of used charges is excluded. Here, we add—in an approximate manner—quantum mechanical effects like exchange to the equation (*E*_ex_ from eq 6) via an additional repulsion component. The electrostatic interaction is thus calculated exactly and is not based on the use of multipole expansions.

Finally, the polarizable density embedding (“PDE”) method can be constructed by a combination of polarizable embedding and density embedding methods. It allows for all features from the density embedding approach, exact electrostatics, and repulsion, combined with polarization effects via dipole–dipole polarizabilities. No self-energy of the embedding environment is included.

### 2.4 Projection-Based Embedding

For projection-based density embedding (“PRE”), the interaction between subsystems is obtained at the low level of theory for the complete system, similar to a subtractive scheme. After a complete DFT calculation, the orbitals are separated into a core and an outer region, each set orthogonal to each other, allowing u to apply operators only on one of these sets.^44−47^ All contributions from eq 6 (except for long-range dispersion) that are described by the applied lower level of theory will automatically be included.

Such a projection-based approach enables high-level calculations on top of the low-level ones. The obtained energy values from this method are always for the complete system and, therefore, include the self-energy of the embedding environment. Our calculation schemes have to change accordingly by subtracting all low-level contributions either of the outer region or of the complete trimer, depending on the type of applied scheme. This method, however, is applicable only after a full system low-level calculation and is thus computationally more demanding than the additive schemes. We will nevertheless apply projection-based embedding to compare with other embedding methods.

### 2.5 Potential-Based Density Embedding

Finally, for the potential density embedding approach, an optimized effective potential (“OEP”) ansatz, where the nonincluded monomers of each subsystem are simulated via an external potential acting on the density, is utilized.^48^ This added external potential, called embedding potential, is responsible for simulating a contribution from the environment onto the region of interest; no environment self-energy is added to the calculation result. The external potential is directly generated by solving an inverse Kohn–Sham problem considering complementary densities from the environment and the complete system density. This allows us to recreate the total system density and should include the most relevant interaction terms, in fact all terms included in eq 6 that the lower level of theory can compute. The method has so far been used to determine the adsorption energies of molecules on surfaces^49^ or the energies of defect states.^50,51^

We consider three possible ways to create external potentials in this method:

- reference a single monomer with a single dimer to the complete trimer (single potential, “POEsi”);
- reference of all monomers to a trimer but also all dimers to the double density of a trimer (separate potentials, “POEdi”);
- reference of all dimers and all monomers to the triple density of the trimer (full, “POEfu”).

A low-level calculation on the full system is needed for this approach, and only the difference between two levels of theory allows a reasonable use; therefore, only subtractive methods will be considered.

## 3 Computational Details

We utilized the generalized gradient approximation (GGA) Perdew–Burke–Ernzerhof (PBE)^52^ functional for the “low level of theory” calculations and the PBE0^53^ hybrid functional as the “high level of theory” method. We chose these two methods because embedding hybrid functionals into GGAs is one of the easiest options to compare different embedding methods. By embedding PBE0 into PBE, the embedding is already similar to existing functionals, such as HSE developed by Scuseria and co-workers,^54^ which uses PBE0 in the short-range term and PBE in the long-range term. The presented data was calculated with the aug-cc-pVTZ basis sets^55^ applying DALTON 2020.0.beta,^56^ MOLPRO 2019.1,^57^ and TURBOMOLE 7.4.1.^58−60^ In the case of VASP (modified VASP.5.4.1^48,61−63^), a 1000 eV energy cutoff and an accurate convergence setting were used. The MOLPRO calculations were performed with density fitting. DFT-based symmetry-adapted perturbation theory (DFT-SAPT) results were obtained from MOLPRO 2019.1 with an aug-cc-pVTZ basis set and density fitting. Except for the DFT-SAPT results, none of the calculations explicitly includes long-range dispersion interactions. Dispersion interaction energies within molecular trimers are nowadays usually added via *a posteriori* corrections to the respective density functional and are subject of other studies.^64,65^ In this work, we focus on induction-induced interactions since commonly used dispersion models^66−76^ can efficiently be applied to complete system calculations, decreasing the impact of embedding on them. Since a metric for measuring the accuracy of the various embedding methods is needed, the errors will be given in reference to the definition for the trimer interaction energy in eq 2 and do therefore not represent the error of the quantum mechanical method (DFT) but rather the error introduced by splitting the system into subsystems. Also, it is important to emphasize again that we are neglecting long-range dispersion. Dispersion-bound systems will have a large short-range dispersion component as well as an electrostatic component coming from higher multipole interactions such as quadrupoles. The former is present in both PBE and PBE0 functionals since they already overestimate interaction energies of hydrogen bonds because of this effect.^77^

## 4 Results and Discussion

**Figure 1:**
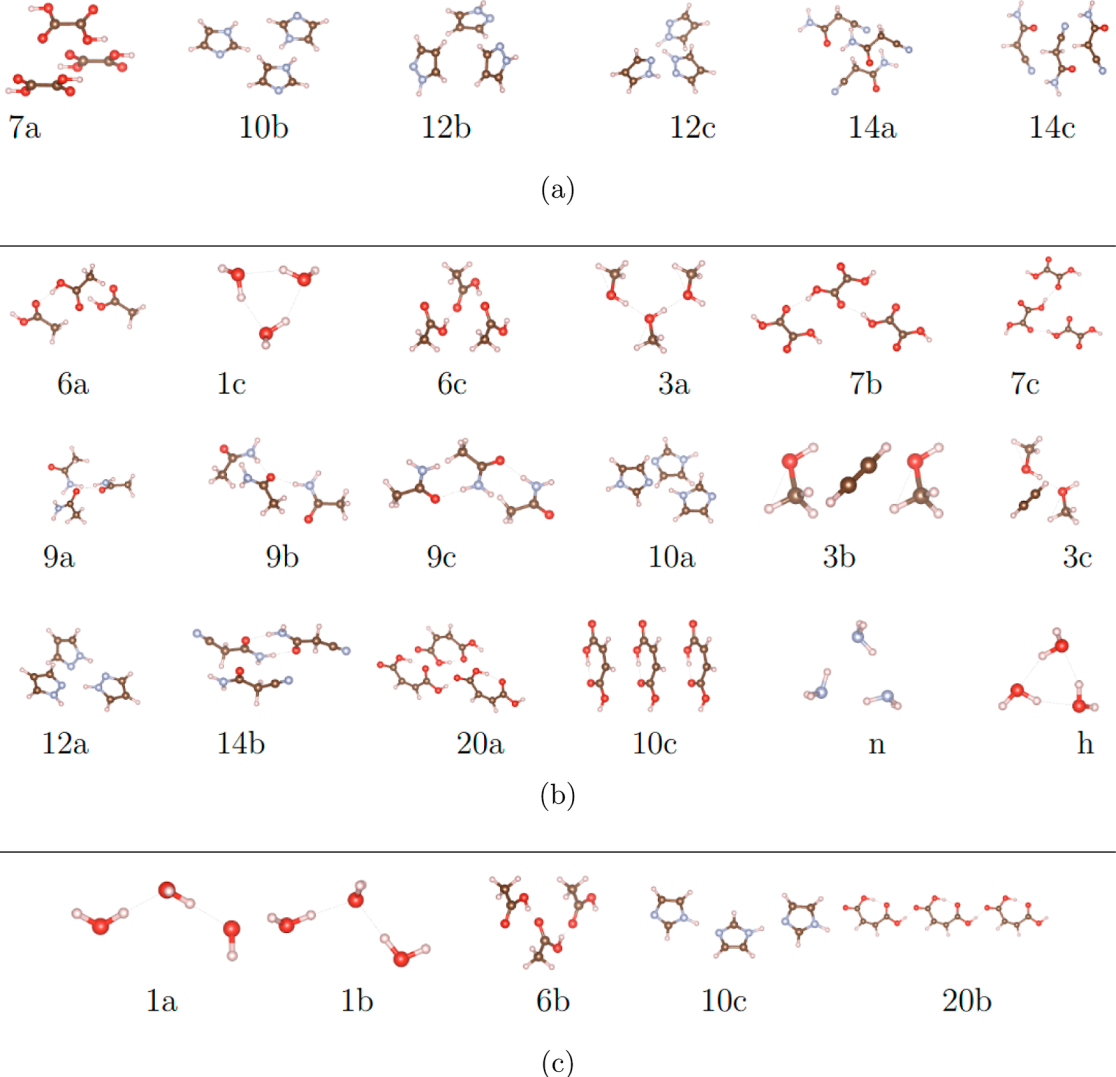
All used trimer structures, ordered according to their *R*_ID_ value. (a) are the dispersion-dominated, (b) are mixed, and (c) are induction-dominated systems.

**Figure 2:**
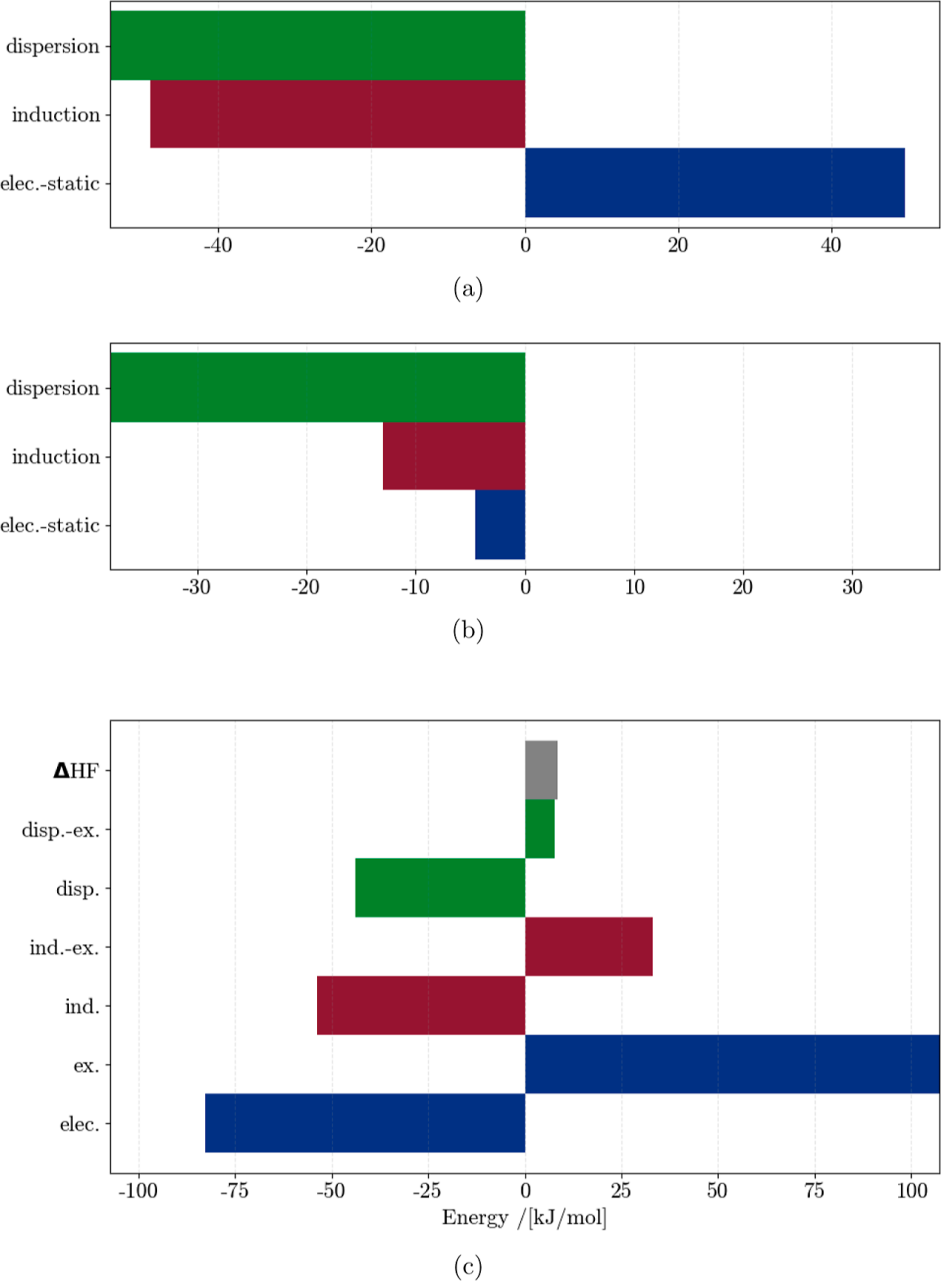
DFT-SAPT results for the induction-dominated trimer 20b (a), the dispersion-dominated trimer 7a (b), and the mixed trimer 6a (c) corresponding to induction and dispersion interaction with their added respective exchange and a trimer with all energy-contributions.

The molecular systems used in this work are listed in Figure 1. The investigated set was mostly taken from the 3B-69 benchmark set^30^ and is composed of small organic molecules. First of all, DFT-SAPT calculations were performed in order to group all of the investigated trimers. Figure 2 shows DFT-SAPT results for three different example trimers. To classify the interactions, we apply two-body SAPT on each included dimer (i.e., three dimers per trimer) and sum up the obtained energy contributions, neglecting nonadditive three-body interactions.

DFT-SAPT gives the following contributions: electrostatic, exchange, induction, induction-exchange, dispersion, dispersion-exchange, and Δ*H*F, whereas the latter is used as an approximation for higher-order induction effects
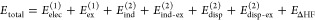
12

The superscript defines the order of perturbation for each contribution, while the subscript defines the physical interpretation.

These can be combined into dispersion, induction, and electrostatic interactions
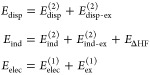
13We found the direct induction *E*_ind_ to dispersion *E*_disp_ ratio
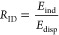
14obtained from SAPT as most instructive when trying to separate dispersion-dominated from induction-dominated systems. A more detailed analysis would investigate the percentage of the mean contribution of a fitted *C*_6_ term in the SAPT dispersion contribution, which is very low for induction-dominated systems.^78^ Systems for which *R*_ID_ < 0.33 are here referred to as “dispersion-dominated” (above first line in Figure 1), whereas trimers with *R*_ID_ > 0.66, are referred to as “dominated by induction” (below second line in Figure 1). In between these two limits, neither induction nor dispersion is dominating, and the systems are labeled as “mixed”. All systems and their *R*_ID_ values are specified in Table S2 in the Supporting Information.

Interaction contributions can be purely additive, like electrostatics, and therefore do not introduce errors if they are summed up. Induction, on the other hand, is highly nonadditive since monomers can screen each other. This screening will not be taken into account in the additive scheme utilized here, introducing substantial errors if the induction is large.

Subtractive schemes do not suffer from these problems as much since induction (i.e., in our case beyond the dimer) is already included via the low-level trimer calculations and the error is only introduced in the difference between low and high levels of theory interaction energy. Of course, this highly depends on the differences between high-level and low-level theories; in case the differences in the interaction energies are large, the errors will be as well. The electrostatic interaction in the trimers of this study generally accounts for a large part of the interaction energy, followed by dispersion and induction. All these three components are substantially counteracted by their corresponding exchange part, depending on the specific system.

Since there are many embedding methods applied, we categorize these into six groups regarding the terms the embedding takes care of as well as the implementation. The groups are

- Unembedded or mechanical embedding (UE).
- Electrostatic embedding, which includes point charge embedding (PCE), dipole charge embedding (DCE), and quadrupole charge embedding (QCE).
- Polarizable embedding, which includes simple polarizable embedding (PE), point charge and polarizable embedding (PPE), dipole and polarizable embedding (DPE), and quadrupole and polarizable embedding (QPE).
- Polarizable density embedding either including a polarizable contribution (PDE) or not (PDEnP).
- Potential-based density embedding (POE) for a monomer in a dimer environment and vice versa (POEsi), for all monomers and all dimers separately (POEdi) and for all monomers and all dimers in a single potential (POEfu).
- Projection-based density embedding (PRE).

**Figure 3:**
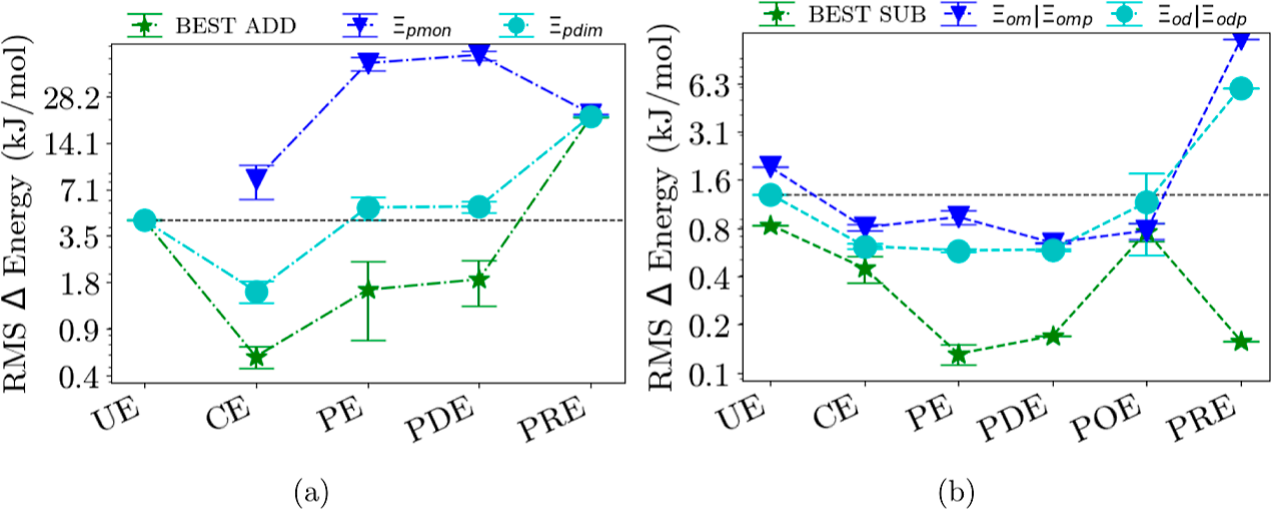
RMS errors of trimer interaction energies Ξ of additive schemes (a) and subtractive schemes (b) for all trimer systems for each embedding method group. Blue triangles denote monomer calculations: Ξ_pmon_, which is Equation S2 for the additive scheme, and Ξ_om_ of eq 5/Ξ_omp_ of eq 8 for the subtractive scheme. Cyan dots mark dimer calculations with exact two-body interactions: Ξ_d_ of eq 3/Ξ_pdim_ of eq 7 for the additive schemes and Ξ_od_ of eq 4/Ξ_odp_ of eq 9 for the subtractive schemes. The green stars show the results obtained with the best-performing scheme for each method.

**Figure 4:**
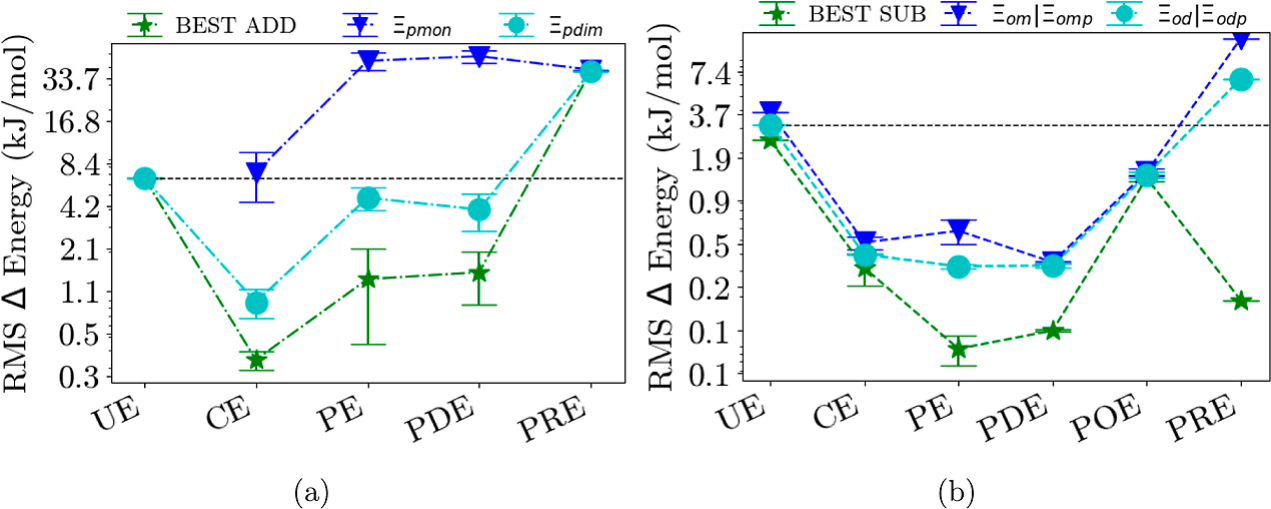
(a) Additive schemes for induction-dominated trimer systems of each embedding method group. (b) Subtractive schemes for induction-dominated trimer systems of each embedding method group; see also the caption of Figure 3. Both can be further separated into monomer (blue) and dimer (cyan) calculations as well as the best-performing scheme (green). All RMS errors are on a logarithmic scale. The dashed line represents the result from the unembedded method. See Supporting Information for a complete figure with all schemes.

**Figure 5:**
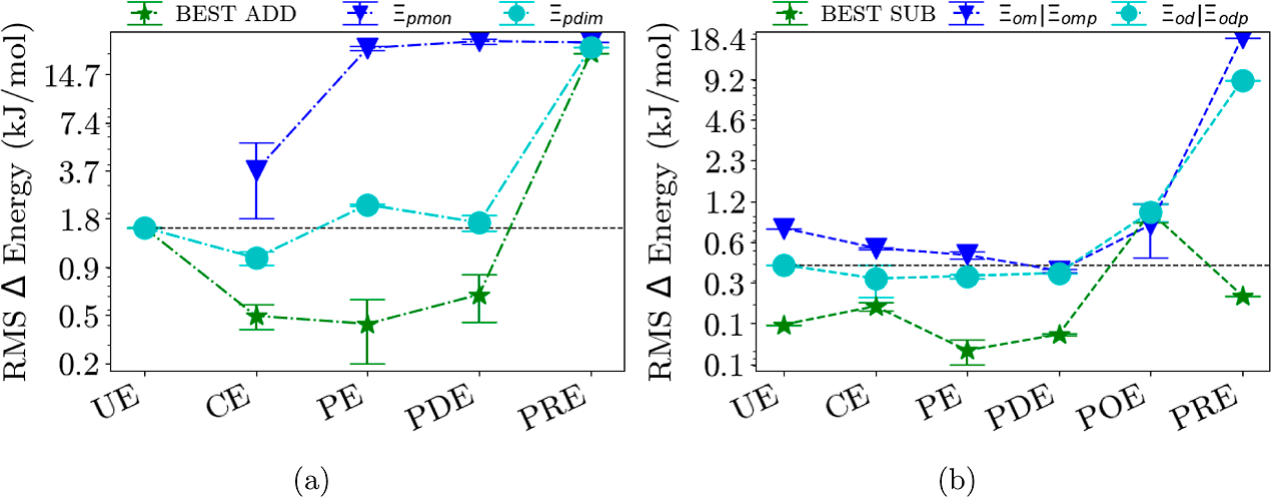
(a) Additive schemes for dispersion-dominated trimer systems of each embedding method group. (b) Subtractive schemes for dispersion-dominated trimer systems of each embedding method group; see also the caption of Figure 3. Both can be further separated into monomer (blue) and dimer (cyan) calculations as well as the best-performing scheme (green). All RMS errors are on a logarithmic scale. The dashed line represents the result from the unembedded method. See Supporting Information for a complete figure with all schemes.

**Figure 6:**
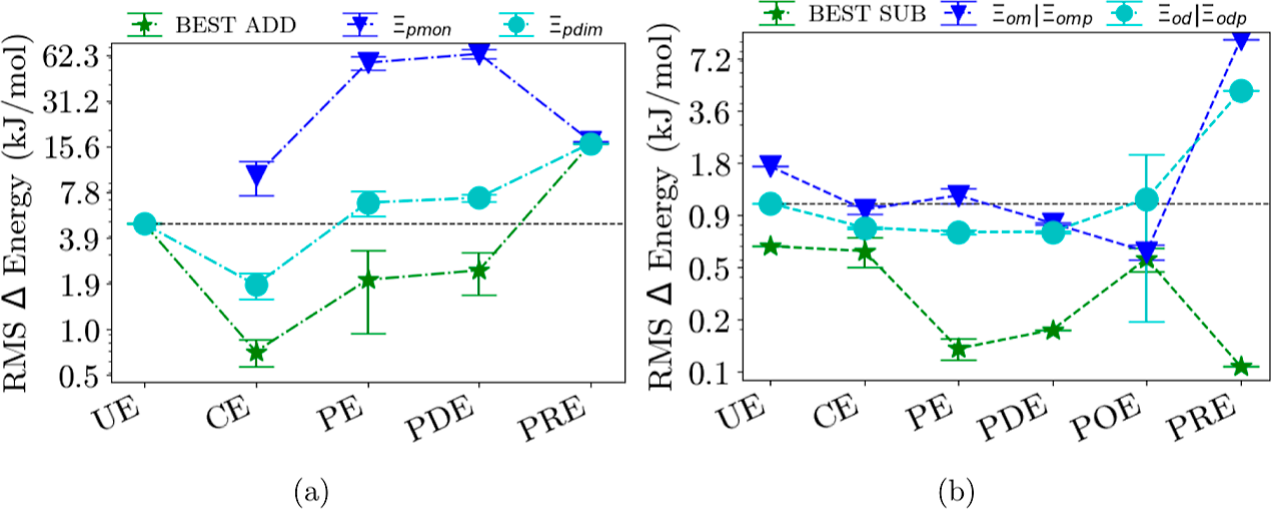
(a) Additive schemes for mixed trimer systems of each embedding method group. (b) Subtractive schemes for mixed trimer systems of each embedding method group, see also the caption of Figure 3. Both can be further separated into monomer (blue) and dimer (cyan) calculations as well as the best-performing scheme (green). All RMS errors are on a logarithmic scale. The dashed line represents the result from the unembedded method. See Supporting Information for a complete figure with all schemes.

Figures 3–6 show each embedding method group separated into additive and subtractive schemes. In addition, monomers or dimers can be calculated at each level to approximate the trimer interaction energy. Also included are the error bars from the different results of members in the group; for example, the CE group includes point charge embedding, dipole embedding, and quadrupole embedding. The uncompressed figures, with each member as a point, are given in the Supporting Information. The potential-based density embedding methods are not included in the additive schemes since they cannot be applied there. The groups are from left to right in ascending or equal levels of theory. We include the overall best scheme results in Figure 3 for better readability; refer to the Supporting Information where more information on the performance of the different methods for various schemes beyond the standard ones is shown.

One may ask the following question: why did we not always choose the optimal scheme for each embedding method into our investigation but discuss the simple standard schemes, even if there may be less error cancellation in these? The answers can be found in the more detailed Figures S20–S23 of the Supporting Information: especially for subtractive schemes with dimers used with density embedding, potential-based density embedding, and projection-based embedding, different schemes yield a surprising variation in results. In particular, the optimal choice is not clear from the start. Using embedding approaches therefore requires careful deliberation of the scheme employed and how to minimize both double-counting effects as well as their error. To compare all methods on the same footing, we employ the exact same schemes for all approaches. In summary, embedding currently does not offer a black-box solution.

All root-mean-square (RMS) errors are on a logarithmic scale. The dashed line represents the result from the unembedded method. See Supporting Information for a complete figure with all schemes.

The additive schemes when comparing the RMS dissociation energies of all trimers yield rather large errors, around 4.81 kJ/mol on the basic dimer level. Using the standard Ξ_pdim_ scheme based on dimers and electrostatic embedding decreases the error to 1.95 kJ/mol. We find the lowest error among the additive schemes for the pd3 scheme (eq 10), which reduces the error further to 0.71 kJ/mol. Interestingly, going beyond electrostatic embedding does not yield much benefit in this respect. Projection-based embedding is inherently derived within a subtractive scheme and thus performs poorly in the additive case.

Finally, the unembedded subtractive method yields a RMS trimer interaction energy error of only 2.25 kJ/mol for the monomer scheme with Equation S2 and 1.61 kJ/mol for the dimer scheme of eq 7, which is considerably lower than the additive ones. In general, the different methods within their category do not differ by much, justifying putting them into different groups.

As could be seen with the additive approaches, a more sophisticated scheme such as from Equation S1 will reduce the error further to 1.13 kJ/mol. While electrostatic embedding reduced the error to 0.92 kJ/mol for the standard scheme of eq 7, there is no improvement for including polarizabilities. However, the combination of electrostatic and polarizability embedding finally reduces the error to as little as 0.28 kJ/mol. For our standard scheme of eq 9, also being the best scheme for this method, this also yields the lowest error over all trimers, thus going beyond the error of the unembedded subtractive scheme by about a factor of 6. For the subtractive schemes, again, most of the different methods within their group category deviate by very little. An exception are the potential-based density embedding methods when utilizing the subtractive schemes; here, the most simple parent method, a monomer in a dimer environment, clearly yields the worst results.

The largest errors and also the reduction of errors can be found for the dissociation energies of the induction-dominated trimer systems in Figure 4. These are usually the hydrogen-bonded systems, and their error is as large as 6.59 kJ/mol for the unembedded additive scheme and 2.46 kJ/mol for the unembedded subtractive scheme. Here, the strength of any embedding method comes into play, and already for electrostatic embedding, the reduction in error is 5.80 kJ/mol, from 6.59 kJ/mol for the unembedded to 0.79 kJ/mol for electrostatic embedding for the additive standard scheme.

For the subtractive standard scheme, the error reduction is significant with a factor of 6 (from 2.46 kJ/mol for the unembedded to 0.37 kJ/mol for the electrostatic embedding). Nevertheless, for the additive as well as subtractive in combination with the standard scheme, including anything else than just electrostatics does not improve the error by much beyond this, which is rather surprising.

Nevertheless, the errors in a subtractive scheme can be reduced for these induction-dominated trimers from 2.46 to 0.06 kJ/mol when using electrostatic and polarizable terms and down to 0.19 kJ/mol for the projection-based density embedding, which is more than 1 order of magnitude. Surprisingly, there is almost no variation when considering the different methods within each group, again justifying grouping them.

In Figure 5, the dispersion-bound systems are investigated in more detail. As the long-range dispersion component is missing from our calculations, electrostatic contributions and possibly short-range dispersion are dominating for these systems.

As one may expect, the errors are considerably lower than those for all systems under investigation in Figure 3. Also, not surprisingly, the combination of the standard schemes does not yield much of an improvement for all embedding methods, on the contrary. For the additive schemes, the error is reduced by electrostatic embedding, and eq 10 is used. From this on, the error can be decreased by polarizable embedding or even further by polarizable embedding with electrostatics.

A similar behavior can be observed for the best subtractive schemes in combination with the different methods. For the subtractive schemes, we observe that the errors stay nearly the same, improving on the embedding theory up to density embedding, confirming the mentioned dominance of electrostatic contributions. It shows that the choice of computation should keep the applied system in mind; a method and scheme that works well in general might have issues with special systems, in this case, dispersion-dominated ones.

Finally, for the mixed systems in Figure 6 using the additive scheme, results look rather similar to those in Figure 5: Electrostatic embedding yields some improvement using the standard scheme compared to the unembedded calculations, whereas all other methods yield worse results. For the best additive scheme, electrostatic embedding yields a considerable improvement of a factor of 9 compared to the unembedded method.

Still, adding polarizable effects or density embedding does not improve the errors. For the subtractive scheme, however, the results are considerably better when any polarizable terms are included in the mix; here, the error is reduced for the standard subtractive schemes from 1.06 (unembedded) to 0.69 kJ/mol for polarizable embedding. Density embedding also gives some improvement, with the best method being potential-based density embedding with an error of 0.44 kJ/mol.

In summary, the introduction of exact two-body interactions decreases errors obtained by the additive schemes well below 2 kJ/mol, whereas only a few of the embedding methods that only included approximations for two-body interactions were able to go below it. Therefore, even the unembedded dimer splitting approach performed better than most monomer embedding approaches, as shown by the black dotted line.

For more insights into the embedding groups as well as many different schemes, see corresponding Section S2 in the appendix. There we provide a detailed overview of which schemes worked best for which method for each of the four groups.

The trivial splitting method results in large errors, representing the neglected interaction energy. There is no monomer-based additive scheme for unembedded systems as it would result in no interaction at all. Using the subtractive scheme, somewhat decent results with overall errors around 1.5 kJ/mol can be achieved, depending on the differences of the two methods applied.

The applied electrostatic embedding methods are computationally cheap and already yield accurate results around 1 kJ/mol, especially when using subtractive schemes. Additive schemes can greatly improve by applying a higher-level embedding, which leads to stable, easy-to-handle, and accurate methods.

Surprisingly, adding higher-order terms does not automatically decrease the introduced error, as we would expect (Figure 3). The error only decreases when additional repulsion effects are added, as seen in the polarizable density embedding method. We observe an error increase if all embedding contributions from polarizable embedding are added up to approximate interaction energies. Only including core region response (electronic as well as nuclear contribution) in all schemes excludes possible double counting and yields lower errors. The computational costs are still rather low, the method performed well in most systems, and the accuracy is high. The additional fraction of time needed for polarizable embedding, in our opinion, is well spent for high accuracy and physically sound interpretations.

**Figure 7:**
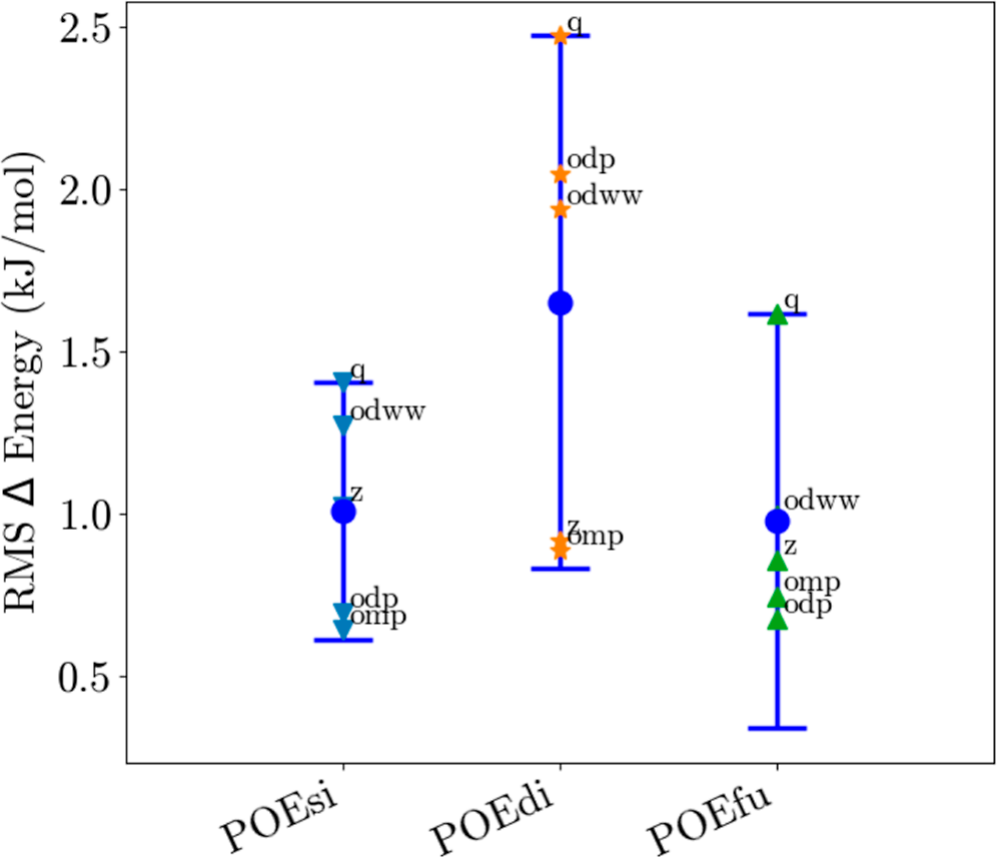
Comparison of the three different approaches of external potential generation, showing the error for all tested ONIOM schemes (see appendix, eqs S8–S15).

The applied OEP methods allow additional approaches for the external potential, as explained in Section 2. It is rather simple to embed each monomer in a dimer to generate the trimer (“POEsi”). All dimers embedded in the same potential (“POEdi”) yield densities that are close to twice the reference and therefore need to be divided by two. All subsystems on the same potential (“POEfu”) result in triple reference densities and therefore need to be divided by three. The necessity of dividing the results by the multiplicity they introduce by OEP creation leads to less control over error cancellation. The errors of the different potential approaches do not drastically differ from each other (see Figure 7); in general, the errors are smaller than those with the mechanical embedding method. However, for each of the three OEP approaches, we find a large variation in RMS with the precise embedding scheme employed (see Supporting Information). While some schemes produce exceedingly accurate results, there are also quite large deviations. In general, the ONIOM schemes provide accurate results. Unfortunately, these methods are rather time-consuming and, at the present time, may not converge easily.

The projection-based density embedding method results in large errors when the standard schemes are used, which do not properly correct for double counting effects. The reasons for these errors are two-fold: (i) the strength of projection-based embedding lies in the proper disentangling of orbitals from different fragments. Since in the present case of molecular trimers there are no covalent bonds to be cut and therefore no significant overlap, this advantage does not apply. (ii) The simple schemes we compare initially lack favorable error cancellation and thus result in comparatively large RMS contributions. Note, however, that the more involved subtraction schemes usually employed with projection-based approaches still result in very accurate predictions (see green symbols in Figure 3b). Like for the OEP methods, the more involved ONIOM schemes result in very small RMS errors (for details, see, e.g., Figure 5 and the RMS tables in the appendix). Applying it in an additive scheme is, as mentioned before, still at the cost of subtractive methods and therefore cannot be recommended. For some of these methods, the subtractive approaches nevertheless work well, leading to one of the lowest errors in this study.

It is worth mentioning that embedding methods are able to generate highly accurate results, but at this point, most of them are rather new and therefore may get optimal performance in the future, decreasing their computational costs. Guided by the results obtained from DFT-SAPT, we expected a large impact on the interaction energy by applying electrostatic embedding with better approximations if we increased the level of theory. This is, however, not always the case. We could indeed observe such a behavior in some cases, but ONIOM-like subtractive schemes, which are already highly accurate, do often not follow this expectation, probably because of error cancellation effects. This difference comes most likely from the slightly different behavior of the high-level and the low-level methods in the embedding schemes, especially since the embedding parameters are, of course, evaluated at the lower level of theory. The density embedding methods including polarizable density embedding, potential-based density embedding, and projection-based density embedding are influenced the most by this effect. All of these methods allow calculation of the reference values also on a higher level of theory, but this in many cases would defy their usefulness.

## 5 Conclusions

Investigating a set of 29 trimer interaction energies, we observe that embedding methods generally improve unembedded approximations for interaction energies. Already, a simple method like electrostatic embedding has a large impact on many systems, especially if the system is known to have high electrostatic contributions such as in induction-based interactions. Beyond electrostatic embedding, the advancement is unfortunately not as large as expected.

In several cases, we even notice an unexpected error increase, rather than an error decrease, because of error cancellation effects when comparing more sophisticated methods to, for example, electrostatic embedding. This also implies that embedding methods need to be improved further when looking at multimer embedding techniques, as in many but not all cases, the error reduction compared to even a mechanical subtractive method is surprisingly small.

Thus, it is very important to realize that there are different possibilities (in this paper, denoted schemes) which can be used in such embedding calculations. In many cases, double-counting occurs, and using these nonstandard schemes reduces the double-counting error. We evaluated several of these schemes, and some combinations appear to be rather promising.

Additive schemes can profit from embedding methods, but errors below 1 kJ/mol, which is close to the RMS threshold of an unembedded subtractive scheme, are extremely rare. Subtractive schemes in combination with embedding methods, on the other hand, have nearly exclusively errors on this order of magnitude and smaller. Unembedded schemes can be improved by a factor of 2 up to a factor of 20 for some embedding methods, resulting in overall errors well below 0.5 kJ/mol. Hence, embedding methods in combination with subtractive schemes are needed to obtain the accurate interaction energies for trimers. This will become especially relevant for a plethora of contemporary approaches toward the calculation of large or periodic systems like molecular crystals, liquids, or high coverage on surfaces. Embedding methods can thus speed up the calculations of trimer interaction energies and provide an avenue beyond raw mechanical embedding for the use of many-body and molecular expansions in these systems.

## Supporting Information Available

List of abbreviations, complete listing of all embedding schemes used, set of molecules, RMS data, method and code combinations, trimer interaction energies, and charges for point charge embedding (PDF)
