# Supplementary material for: Dissociation Energies via Embedding Techniques
Source: J Phys Chem A. 2024 Oct 15;128(42):9275–86. doi: 10.1021/acs.jpca.4c02851 (PMC11514013; doi:10.1021/acs.jpca.4c02851)
Supplement: Supplementary file 1 — jp4c02851_si_001.pdf [file jp4c02851_si_001.pdf]

Supplemental Information: Dissociation Energies via Embedding Techniques

Florian Feyersinger,[†,‡] Peter E. Hartmann,[†] Johannes Hoja,[†] Peter Reinholdt,[¶] Florian Libisch,[§] Jacob Kongsted,[‖] Peter Puschnig,[‡] and A. Daniel Boese[*,†]

†*Department of Chemistry, University of Graz, Heinrichstraße 28/IV, 8010 Graz, Austria.*

‡*Department of Physics, University of Graz, 8010 Graz, Austria.*

¶*Department of Physics, Chemistry and Pharmacy, University of Southern Denmark, Campusvej 55, 5230, Odense M, Denmark*

§*Institute for Theoretical Physics, Vienna University of Technology.*

‖*Department of Physics, Chemistry and Pharmacy, University of Southern Denmark.*

E-mail: adrian_daniel.boese@uni-graz.at

Contents

S1 Glossary **S3**

S2 Schemes **S12**

S2.1 Schemes for mechanical embedding S12

S2.2 Additive Schemes for Embedded Subsystems S15

S2.3 Subtractive Schemes/ONIOM like Approaches for Embedded Subsystems . . S19

S3 Set of Molecules **S31**

S3.1 DALTON S33

S4 RMS Tables **S47**

S5 Method and code combinations **S51**

S6 Trimer Interaction Energies PBE0 vs. PBE **S51**

S7 Charges for point charge embedding **S52**

References **S53**

S1 Glossary

We realize that this paper includes a lot of abbreviations can lead to confusion. If the descriptions is not done in a few words you can find a thorough description of each abbreviation elsewhere, this glossary will help you find it. Note that numbered equations or sections in the supplement are preceeded by the letter S, e.q., Equation S1, while a corresponding number without S refers to an aquation in the manuscript.

- **E_{inter}** interaction energy approximation via subsystems,section 1, Equation 1
- **QM** quantum mechanic
- **MM** molecular mechanic
- **DFT-SAPT** Density Functional Theory Symmetry Adapted Perturbation Theory, section 4
- **GGA** Generalized Gradient Approximation, in this paper the low level of theory
- Ξ Trimer Interaction Energy,section 1, 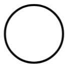,Equation 2 and section S2 Figure S1
- DIME Dimer Interaction Energy,
- Ξ_d Trimer Interaction Energy with dimer approximation,subsection 2.1, 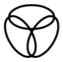,Equation 3 and section S2 Figure S2
- **ONIOM** Our Own N-layered integrated molecular orbital mechanics, subtractive scheme,subsection 2.1

- Ξ_{om} Ξ with ONIOM approach at monomer level,subsection 2.1, 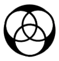 ,Equation 5 and section S2 Figure S3

- Ξ_{od} Ξ with ONIOM approach at dimer level,subsection 2.1, 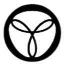 ,Equation 4 and section S2 Figure S4

- Ξ_{odw} Ξ with ONIOM approach at dimer level only accounting for interaction, 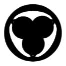 ,Equation S1 and section S2 Figure S5

- embedded obtained from an embedding method

- high obtained at high level of theory, in this paper at hybrid functional level(PBE0),section 3

- low obtained at low level of theory, in this paper at GGA functional level (PBE),section 3

- **UE** simple calculation of the corresponding system no embedding applied, mechanical embedding,unembedded,subsection 2.1

- E_{int} interaction energy defined by its contributions,subsection 2.1 ,Equation 6

- $\mathbf{E}_{\mathrm{pc}}$ point charge interaction energy contribution,subsection 2.1 ,Equation 6

- $\mathbf{E}_{dip}$ dipole interaction energy contribution,subsection 2.1 ,Equation 6

- $\mathbf{E}_{\mathrm{quad}}$ quadrupole interaction energy contribution,subsection 2.1 ,Equation 6

- $\mathbf{E}_{\mathrm{mult}}$ multipole (higher orden then quadrupole) interaction energy contribution,subsection 2.1 ,Equation 6

- $\mathbf{E}_{\text{indu}}$ induction interaction energy contribution,subsection 2.1 ,Equation 6
- $\mathbf{E}_{\text{dispe}}$ dispersion interaction energy contribution,subsection 2.1 ,Equation 6
- $\mathbf{E}_{\text{exc}}$ exchange interaction energy contribution,
- **PCE** point charge embedding via TURBOMOLE,subsection 2.2, section 4, Figure 3 to Figure 4,Figure S20 to Figure S21
- **DCE** dipole embedding vie DALTON,subsection 2.2 ,section 4, Figure 3 to Figure 4,Figure S20 to Figure S21
- **QCE** quadrupole embedding via DALTON,subsection 2.2 ,section 4, Figure 3 to Figure 4,Figure S20 to Figure S21
- **PE** polarizable embedding via DALTON,subsection 2.2 ,section 4, Figure 3 to Figure 4,Figure S20 to Figure S21
- **CC** Coupled Cluster
- **PPE** point charge combined with polarizable embedding via DALTON,subsection 2.2 ,section 4, Figure 3 to Figure 4,Figure S20 to Figure S21
- **DPE** dipole combined with polarizable embedding via DALTON,subsection 2.2 ,section 4, Figure 3 to Figure 4,Figure S20 to Figure S21
- **QPE** quadrupole combined with polarizable embedding via DALTON,subsection 2.2 ,section 4, Figure 3 to Figure 4,Figure S20 to Figure S21
- **PDEnP** polarizable Density embedding without polarizability contribution via DALTON,subsection 2.3 ,section 4, Figure 3 to Figure 4,Figure S20 to Figure S21

- **PDE** polarizable Density embedding via DALTON,**??** ,section 4, Figure 3 to Figure 4,Figure S20 to Figure S21

- **PRE** projection-based density embedding via MOLPRO,subsection 2.4 ,section 4, Figure 3 to Figure 4,Figure S20 to Figure S21

- **OEP** Optimized External Potential method,subsection 2.5

- **POEsi** potential based density embedding via VASP with a single potential for a dimer and a monomer,subsection 2.5, section 4, Figure 3 to Figure 4,Figure S20 to Figure S21

- **POEdi** potential based density embedding via VASP with one potential for monomers and one for dimers,subsection 2.5, section 4, Figure 3 to Figure 4,Figure S20 to Figure S21

- **POEfu** potential based density embedding with all subsystem on a single potential,subsection 2.5, section 4, Figure 3 to Figure 4,Figure S20 to Figure S21

- R_{ID} relative induction to dispersion energy obtained from DFT-SAPT,section 4, Equation 14

- $\mathbf{E}_{\mathrm{total}}$ total energy from DFT-SAPT,section 4, Equation 14, Equation 4, Equation 13

- $\mathbf{E}_{\mathrm{ind}}$ induction energy from DFT-SAPT,section 4, Equation 14, Equation 4, Equation 13

- $\mathbf{E}_{\mathrm{ind.-ex}}$ induction exchange part from DFT-SAPT,section 4, Equation 14, Equation 4, Equation 13

- **$\mathbf{E}_{\Delta \mathrm{HF}}$** higher order induction terms from DFT-SAPT, Equation 14, Equation 4, Equation 13

- **$\mathbf{E}_{\mathrm{disp}}$** dispersion energy from DFT-SAPT,section 4, Equation 14, Equation 4, Equation 13

- **$\mathbf{E}_{\mathrm{disp.-ex}}$** dispersion exchange term from DFT-SAPT,section 4, Equation 14, Equation 4, Equation 13

- **$\mathbf{E}_{\mathrm{elec}}$** electrostatic contribution in DFT-SAPT,section 4, Equation 4, Equation 13

- **BEST ADD.** best additive schemes applied, may differ between methods, section 4,Figure S20 to Figure S21 and section S4

- Ξ_{pmon} Ξ on embedded monomer level, 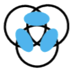,Equation S2 and section S2 Figure S6

- Ξ_{pdim} Ξ on embedded dipole level,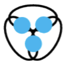,Equation S4 and section S2 Figure S8

- **BEST SUB.** best subtractive/ONIOM scheme applied, may differ between methods, section 4,Figure S20 to Figure S21 and section S4

- Ξ_{omp} Ξ at ONIOM level with monomer subsystems,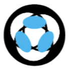,Equation S8 and section S2 Figure S12

- Ξ_{odp} Ξ at ONIOM level with dimer subsystems,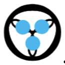,Equation S9 and section S2 Figure S13

- Ξ_{z} a version of Ξ at ONIOM level,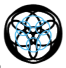,Equation S14 and section S2 Figure S18

- Ξ_{pd3} a version of Ξ at ONIOM level, 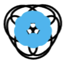,Equation S7 and section S2 Figure S11

- **elec.** electronic contribution in dalton (embedding) approaches

- **nuc.** nuclear contribution in dalton (embedding) approaches

- **rep.** repulsion contribution in dalton (embedding) approaches

We have also mentioned various calculation schemes and provide here a table with their abbreviations, symbols and where to find them/where they are mentioned.

Table S1: All schemes their symbol and where to find them

Scheme	Symbol	Reference	Short
Ξ	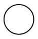	Equation 2 Figure S1	trimer interaction energy
Ξ_{d}	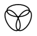	Equation 3 Figure S2	trimer interaction energy on dimer level
Ξ_{om}	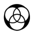	Equation 5 Figure S3	subtractive trimer energy on monomer level
Ξ_{od}	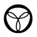	Equation 4 Figure S4	subtractive trimer energy on dimer level
Ξ_{odw}	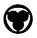	Equation S1 Figure S4	subtractive trimer interaction energy on dimer level
Ξ_{pmon}	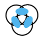	Equation S2 Figure S6	trimer interaction energy at dimer embedded monomer level
Ξ_{pm2}	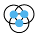	Equation S3 Figure S6	trimer interaction energy at monomer embedded monomer level
Ξ_{pdim}	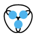	Equation S4 Figure S8	trimer interaction energy at embedded dimer level
Ξ_{pd2}	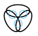	Equation S5 Figure S9	trimer interaction energy at monomer embedded monomer corrected embedded dimer level
Ξ_{pd3}	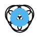	Equation S7 Figure S6	trimer interaction energy at dimer embedded monomer corrected embedded dimer level
Ξ_{omp}	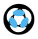	Equation S8 Figure S12	subtractive trimer energy at dimer embedded monomer level
Ξ_{odp}	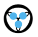	Equation S9 Figure S13	subtractive trimer energy at embedded dimer level
Ξ_{omp2}	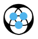	Equation S10 Figure S14	subtractive trimer energy at monomer embedded monomer level
Ξ_{omp3}	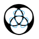	Equation S11 Figure S15	subtractive trimer energy at monomer embedded monomer corrected embedded monomer level
Ξ_{y}	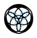	Equation S12 Figure S16	subtractive trimer energy at monomer embedded monomer corrected embedded dimer level
Ξ_{z}	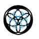	Equation S14 Figure S18	subtractive trimer interaction energy at dimer embedded monomer corrected embedded dimer level
Ξ_{odww}	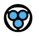	Equation S13 Figure S17	subtractive trimer interaction energy at embedded dimer level

In Table S2, we show a short table of energy contributions in terms of monomers and interactions included in the various groups of embedding approaches.

- UE implies the unembedded method
- CE charge embedding
- PE polarizable embedding
- PDE/DE polarize density embedding/Density embedding
- POE potential embedding
- PRE projection-based embedding

For our evaluation, we need to combine a method with a certain scheme of Table S1.

For example in Table S2, *PE monomer A embedded monomer embedded B* includes an active monomer A which is embedded by a polarizable force field into monomer B, thus containing an active monomomer A and an embedded interaction to monomer B.

Table S2: Energy contributions of used methods, ^{active} denotes the system that sees the embedding area whereas ^{embed} denotes the area of interest presented with the embedding approach. $_{monomerX}$ is the unperturbed Energy of monomer X, $_{interactionXY(Z)}$ is the perturbation of unperturbed monomers X,Y and potentially Z representing an interaction. * only ONIOM like approaches available

Method group	Calculation type	Contributions
UE	monomerA	$E^{active}_{monomerA}$
UE	dimerAB	$E^{active}_{monomerA}$,$E^{active}_{monomerB}$,$E^{active}_{interactionAB}$
UE	ONIOM dimerAB by monomers	$E^{active}_{low,monomerA}$,$E^{active}_{low,monomerB}$,$E^{active}_{low,interactionAB}$, $E^{active}_{high,monomerA}$,$E^{active}_{high,monomerB}$
UE	trimerABC	$E^{active}_{monomerA}$,$E^{active}_{monomerB}$,$E^{active}_{monomerC}$, $E^{active}_{interactionABC}$
CE	monomerA embedded monomerB	$E^{active}_{monomerA}$, $E^{embed}_{interactionAB}$
CE	dimerAB embedded monomerC	$E^{active}_{monomerA}$,$E^{active}_{monomerB}$, $E^{active}_{interactionAB}$,$E^{embed}_{interactionABC}$
PE	monomerA embedded monomerB	$E^{active}_{monomerA}$, $E^{embed}_{interactionAB}$
PE	dimerAB embedded monomerC	$E^{active}_{monomerA}$,$E^{active}_{monomerB}$, $E^{active}_{interactionAB}$,$E^{embed}_{interactionABC}$
PDE/DE	monomerA embedded monomerB	$E^{active}_{monomerA}$, $E^{embed}_{interactionAB}$
PDE/DE	dimerAB embedded monomerC	$E^{active}_{monomerA}$,$E^{active}_{monomerB}$, $E^{active}_{interactionAB}$,$E^{embed}_{interactionABC}$
POE	ONIOM* monomerA embedded monomerB	$E^{active}_{low,monomerA}$,$E^{active}_{high,monomerA}$, $E^{active}_{low,monomerB}$,$E^{active}_{high,monomerB}$, $E^{embed}_{high,interactionAB}$, $E^{active}_{low,interactionAB}$
POE	ONIOM* dimerAB embedded monomerC	$E^{active}_{low,monomerA}$,$E^{active}_{high,monomerA}$, $E^{active}_{low,monomerB}$,$E^{active}_{high,monomerB}$, $E^{active}_{low,monomerC}$,$E^{active}_{high,monomerC}$, $E^{embed}_{high,interactionABC}$, $E^{active}_{low,interactionABC}$, $E^{active}_{low,interactionAB}$,$E^{active}_{high,interactionAB}$
PRE	monomerA embedded monomerB	$E^{active}_{monomerA}$,$E^{embed}_{interactionAB}$, $E^{active}_{low,dimerAB}$
PRE	dimerAB embedded monomerC	$E^{active}_{monomerA}$,$E^{active}_{monomerB}$, $E^{embed}_{interactionABC}$, $E^{active}_{low,trimerABC}$

S2 Schemes

S2.1 Schemes for mechanical embedding

We defined the trimer interaction as mentioned in the main article via Equation 2. Here, filled (black) circles represent the low level of theory, while open (white) circles will represent a high-level approach below.

We also introduce a compact/verbal version of the notation in the main article, all equations not written in the main article will have the compact notation in the corresponding schematic. Here we neglect the "total" superscript and write the monomer and dimer sums as $\sum^{\text{trimer}} E_{\text{monomers}}$ and $\sum^{\text{trimer}} E_{\text{dimers}}$ respectively.

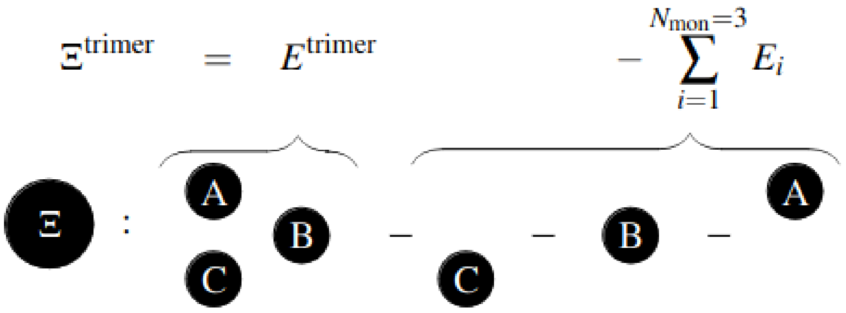

Figure S1: Schematic of Equation 2. Black encircled A, B and C represent different monomers

Since unembedded monomers are not able to fulfill an additive scheme, we instantly start with dimer subsystems resulting in a trimer interaction energy at the dimer level Figure S2, white circles representing a high level of theory.

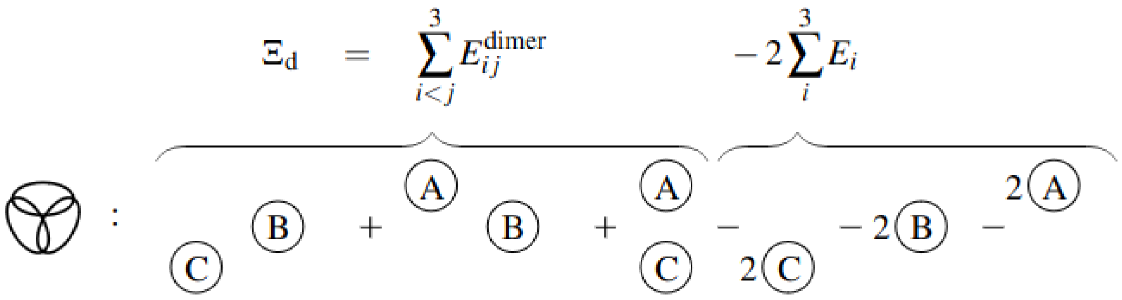

Figure S2: Schematic of Equation 3. Black encircled A, B and C represent different monomers

The corresponding subtractive scheme would include the same subsystems as the additive scheme, resulting in Figure S4 and Figure S3. We subtract the high level monomers in Figure S4 since in a step between we obtain the high level full trimer approximation.

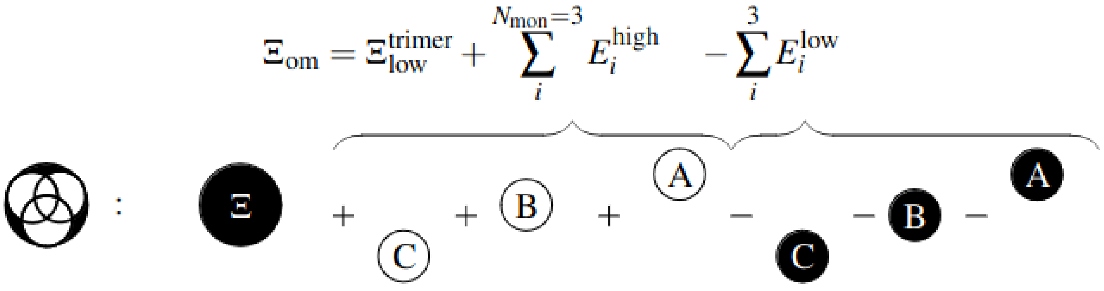

Figure S3: example Schematic of Equation 5 with monomers as sub-systems. Black A, B and C represent different monomers at low-level of theory. Encircled A, B and C show monomers in high-level of theory

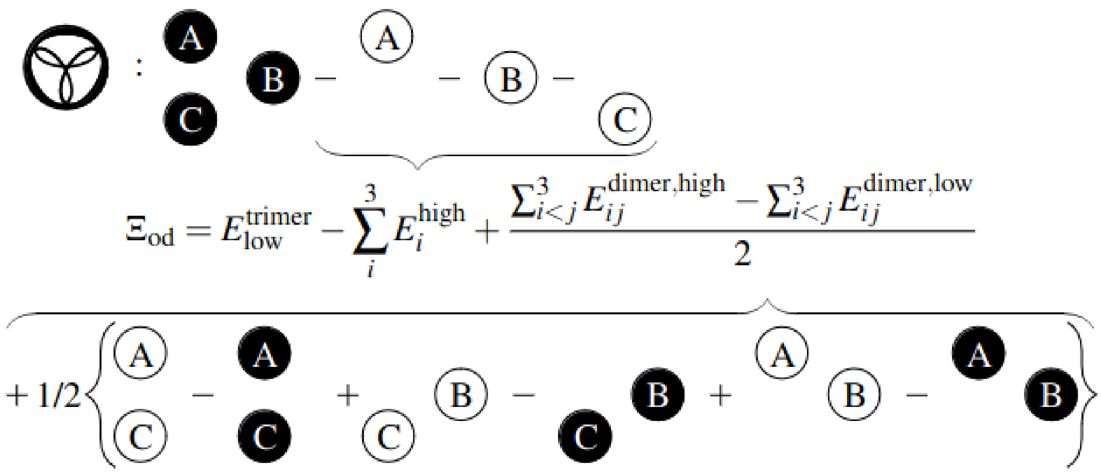

Figure S4: example Schematic of Equation 4 with monomers as sub-systems. Black A, B and C represent different monomers at low-level of theory. Encircled A, B and C show monomers in high-level of theory

For Figure S5, we are not interested in obtaining the full high-level trimer anymore. We add the difference of dimer interaction of the two levels of theory and add it to the low-level trimer. It is obvious that with this scheme, we end up with a low-level trimer including a high-level trimer interaction energy approximation.

$$\begin{aligned}\Xi_{\text{odw}} =& \Xi^{\text{low}} + \sum^{\text{trimer}} E_{\text{dimers}}^{\text{high}} - 2\sum^{\text{trimer}} E_{\text{monomers}}^{\text{high}} \\ &- \sum^{\text{trimer}} E_{\text{dimers}}^{\text{low}} + \sum^{\text{trimer}} E_{\text{monomers}}^{\text{low}}\end{aligned} \tag{S1}$$

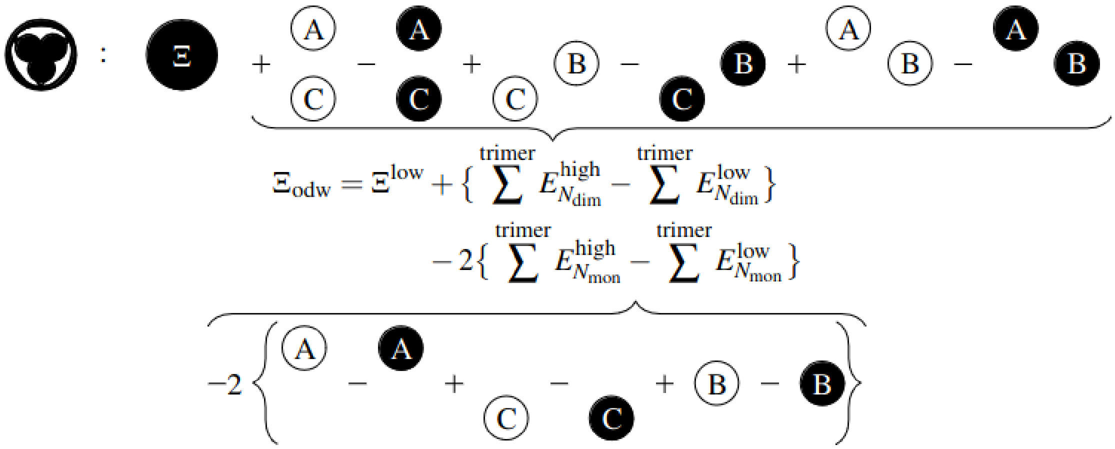

Figure S5: example Schematic of Equation 4. Black A, B and C represent different monomers at low-level of theory. Encircled A, B and C show monomers in high-level of theory

S2.2 Additive Schemes for Embedded Subsystems

Finishing up the unembedded schemes, we now start to introduce embedding, visualized in cyan.

Similar approaches as in the unembedded case can be done, this time we start with the monomer subsystems:

$$\Xi_{\text{pmon}} = \sum^{\text{trimer}} E^{\text{embedded}-\text{dimers}}_{\text{monomers}} - \sum^{\text{trimer}} E_{\text{monomers}} \tag{S2}$$

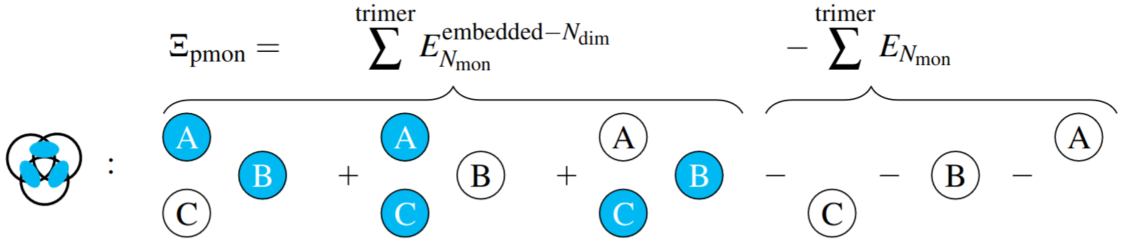

Figure S6: Schematic of Equation S2. encircled A, B and C represent different monomers. Cyan A, B and C show the embedded monomers.

The equation and scheme above gives the approximation for the trimer interaction energy dependent on the embedding influence of embedded dimers on the monomer subsystems. Like

all additive schemes, it can give results for any level of theory.

Now things can get complicated, as the newly added superscript "embedded − dimers" represents the size of the environment/the counter subsystem/the embedded region of the obtained energy. The example embedded − dimers therefore means that the monomer is embedded in a dimer environment. Another superscript will be "embedded − monomers", indicating embedding in a monomer subsystem, the dimers with the embedded superscript will always be embedded in a monomer.

Since a monomer may be embedded in two different other monomers inside a trimer system, we need to sum over all possible permutations. This will later be marked as $\sum^{\text{trimer}}$, implying six systems for monomer in monomer embedding and three for every other case.

$$\Xi_{\text{pm2}} = \sum^{\text{trimer}} E_{\text{monomers}}^{\text{embedded-monomers}} - 2 \sum^{\text{trimer}} E_{\text{monomers}} \tag{S3}$$

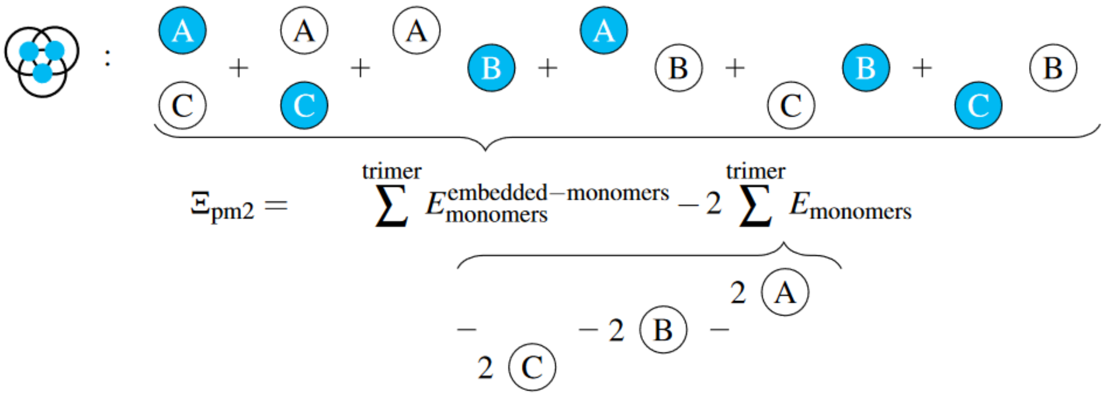

Figure S7: Schematic of Equation S3. encircled A, B and C represent different monomers. Cyan A, B and C show the embedded monomers.

For Equation S3, we did not have to divide the result by two, since we only count each monomer interaction energy once. In case of Equation S4, we have a sum over all embedding dimers that lead to each monomer appearing twice and each interaction with the other monomers once fully and once with the embedding approximation. This is indeed a problem, and the trimer interaction obtained needs to be divided by two. In the following,

we will devise several schemes to counteract this.

$$\Xi_{\mathrm{pdim}} = \frac{\sum^{\mathrm{trimer}} E^{\mathrm{embedded}}_{\mathrm{dimers}} - 2\sum^{\mathrm{trimer}} E_{\mathrm{monomers}}}{2} \tag{S4}$$

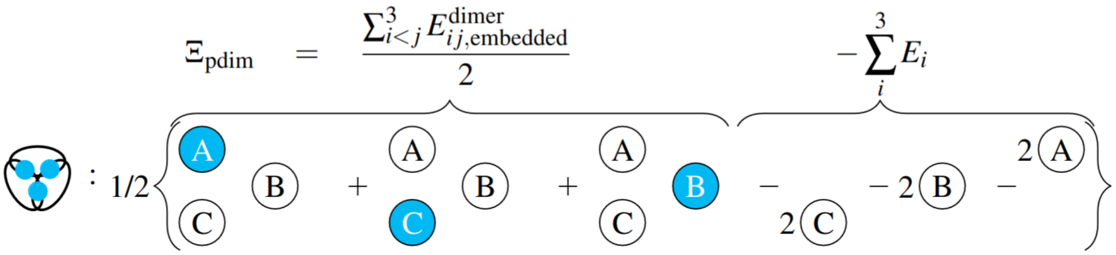

Figure S8: Schematic of Equation S4. encircled A, B and C represent different monomers. Cyan A, B and C show the embedded monomers.

The schemes for embedding are straight forward and similar to the non-embedded approach so far.

Yet, embedding allows not only to devise these schemes, but we will try to include and exclude various influences of embedding (for example, point charges) on the final result. Unfortunately, we need to use different approaches to counteract double counting of the embedding contributions!

The previously mentioned Ξ_{pdim} scheme could be expanded to a scheme that does include the embedding interaction on a two-body subsystem, but reduces double-counting, removing monomer in monomer embedding interactions. This would result in Equation S5, and somewhat counteracts the issue of having to divide the obtained interaction by two.

$$\Xi_{\mathrm{pd2}} = \sum^{\mathrm{trimer}} E^{\mathrm{embedded}}_{\mathrm{dimers}} - \sum^{\mathrm{trimer}} E^{\mathrm{embedded-monomers}}_{\mathrm{monomers}} \tag{S5}$$

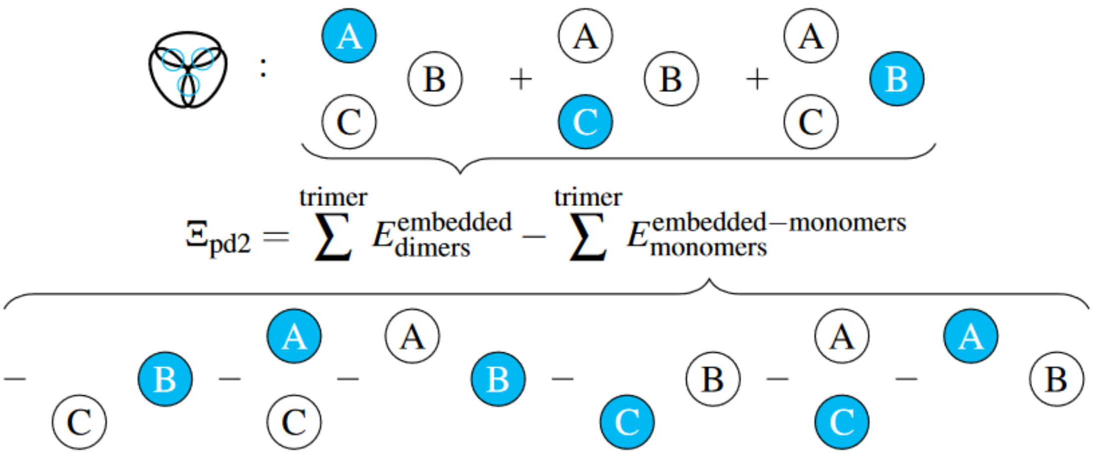

Figure S9: Schematic of Equation S5. encircled A, B and C represent different monomers. Cyan A, B and C show the embedded monomers.

The same can be done for a monomer subsystem embedded in the dimer environment, giving Equation S6. Unfortunately, since there are six different embedded monomers, we have to count all monomer in dimer systems twice and therefore again have to divide the obtained result by two.

$$\Xi_{pm3} = \frac{2\sum^{trimer} E^{embedded-dimers}_{monomers} - \sum^{trimer} E^{embedded-monomers}_{monomers}}{2} \tag{S6}$$

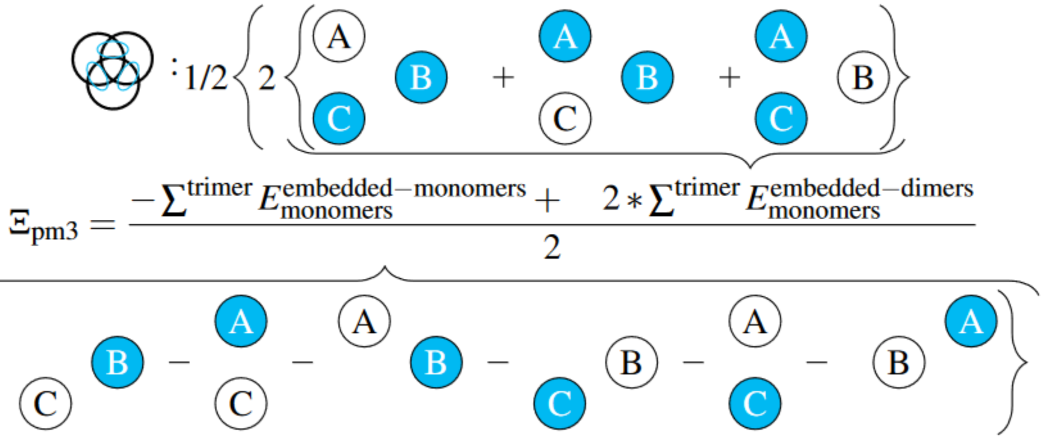

Figure S10: Schematic of Equation S6. encircled A, B and C represent different monomers. Cyan A, B and C show the embedded monomers.

In Equation S5, we excluded the embedded interaction of monomers in other monomers,

but it would also be possible to exclude only the monomer to the dimer embedded interaction. This should counteract double counting on the three body interaction level and only sums over three subsystems. The new equation is given in Equation S7 and is indeed one of the most successful schemes we applied in this work.

$$\Xi_{\text{pd3}} = \sum^{\text{trimer}} E^{\text{embedded}}_{\text{dimers}} - \sum^{\text{trimer}} E_{\text{monomers}} - \sum^{\text{trimer}} E^{\text{embedded}-\text{dimers}}_{\text{monomers}} \tag{S7}$$

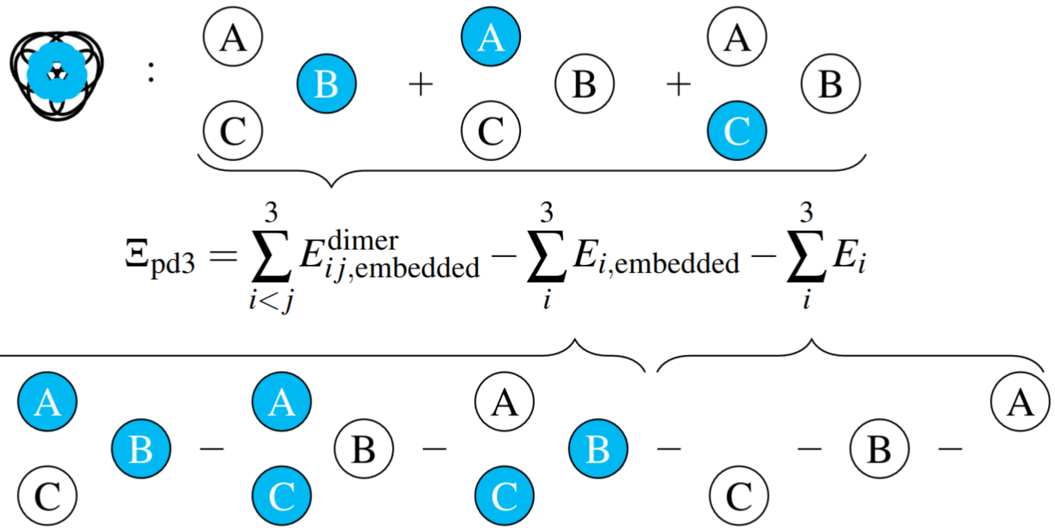

Figure S11: Schematic of Equation S7. encircled A, B and C represent different monomers. Cyan A, B and C show the embedding monomers.

S2.3 Subtractive Schemes/ONIOM like Approaches for Embedded Subsystems

To include low level trimer interaction and only approximate high level of theory trimer interaction, we again apply subtractive schemes.

Easy accessible are Equation S8, Equation S9 and Equation S10 each corresponding to a specific kind of subsystem-embedding approach and therefore only including one type of interaction approximation. We have seen for additive schemes that we can in/exclude specific interaction contributions, reducing double-counting effects. This is leading to even more different schemes, possibly improving their interaction energy.

Equation S8 is the basic approach of adding simulated embedding trimer energy difference to a low-level trimer and to obtain an approximation of the complete high-level trimer.

$$\Xi_{\text{omp}} = \Xi^{\text{low}} + \sum^{\text{trimer}} E_{\text{monomers}}^{\text{embedded,high}} - \sum^{\text{trimer}} E_{\text{monomers}}^{\text{embedded,low}} \tag{S8}$$

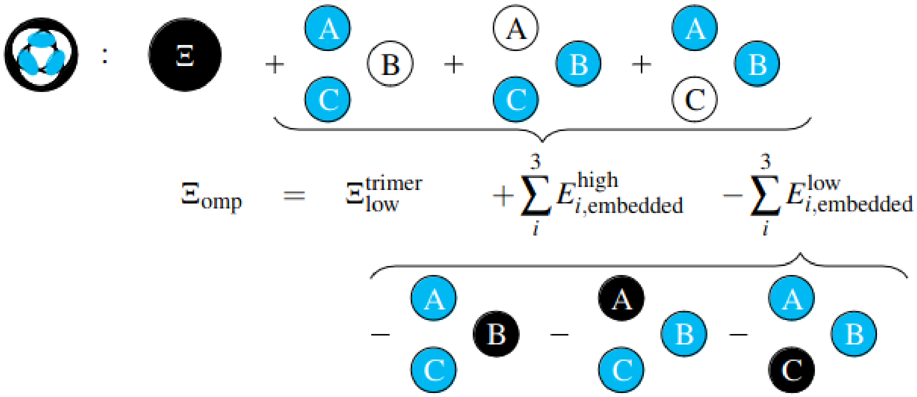

Figure S12: example Schematic of Equation S8. Black A, B and C represent different monomers at low-level of theory. Encircled/white A, B and C show monomers in high-level of theory. Cyan A, B and C show the embedding monomers.

We can use Equation S9 to obtain a high-level approximation of the full trimer using embedded dimers. In this scheme, we divide by two to counteract the double counting of the monomer energies.

$$\Xi_{\text{odp}} = \Xi^{\text{low}} + \frac{\sum^{\text{trimer}} E_{\text{dimers}}^{\text{embedded,high}} - \sum^{\text{trimer}} E_{\text{dimers}}^{\text{embedded,low}}}{2} \tag{S9}$$

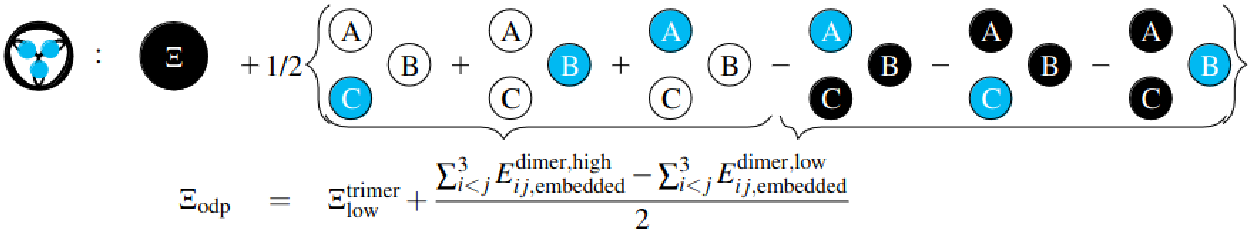

Figure S13: example Schematic of Equation S9. Black A, B and C represent different monomers at low-level of theory. Encircled/white A, B and C show monomers in high-level of theory. Cyan A, B and C show the embedded monomers.

The identical approach leads in the case of monomer in monomer embedding to Equation S10.

$$\Xi_{omp2} = \Xi^{low} + \frac{\sum^{trimer} E_{monomers}^{embedded-mon,high} - \sum^{trimer} E_{monomers}^{embedded-mon,low}}{2} \tag{S10}$$

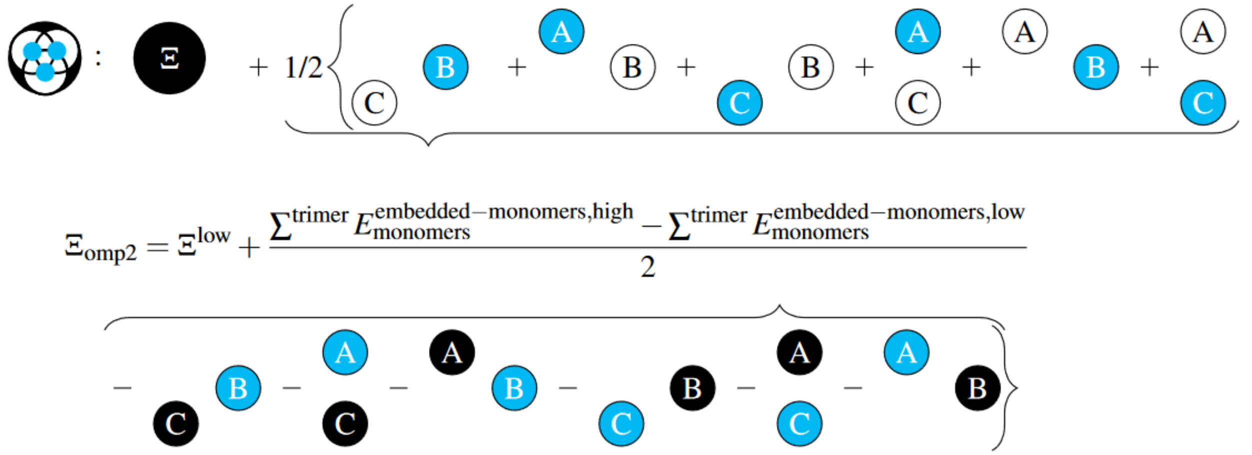

Figure S14: Schematic of Equation S10. Black A, B and C represent different monomers at low-level of theory. Encircled/white A, B and C show monomers in high-level of theory. Cyan A, B and C show the embedded monomers.

Again, we can counteract various embedding interactions, leading to further schemes: In Equation S11, we sum over all monomer embedding methods and add the embedded trimer interaction energy difference to the exact low level of theory. We are subtracting

the embedded dimer interaction energy difference to counteract various double counting errors. This can be seen as an subtractive version of a combination between Equation S2 and Equation S3.

$$\begin{aligned}\Xi_{\text{omp3}} = &\Xi^{\text{low}} + \\ &\frac{2\sum^{\text{trimer}} E_{\text{monomers}}^{\text{embedded}-\text{dimers,high}} - 2\sum^{\text{trimer}} E_{\text{monomers}}^{\text{embedded}-\text{dimers,low}}}{2} \\ &+ \frac{-\sum^{\text{trimer}} E_{\text{monomers}}^{\text{embedded}-\text{monomers,high}} - \sum^{\text{trimer}} E_{\text{monomers}}^{\text{embedded}-\text{monomers,low}}}{2}\end{aligned} \tag{S11}$$

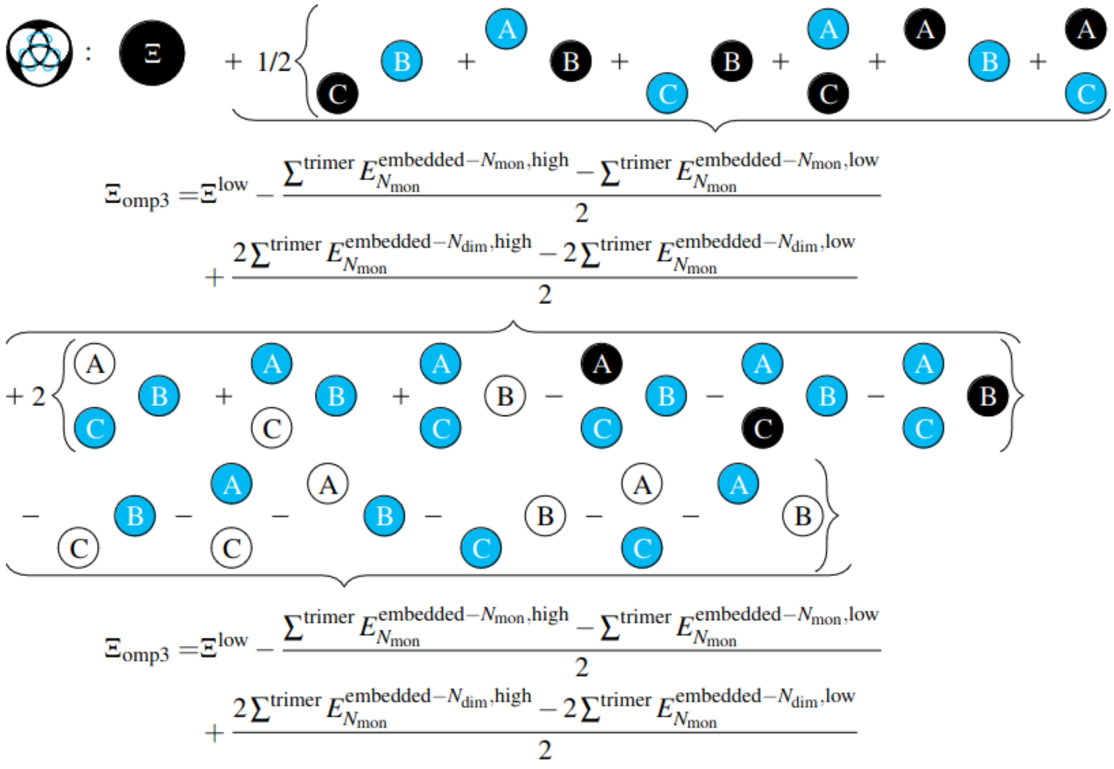

Figure S15: Schematic of Equation S11. Black A, B and C represent different monomers at low-level of theory. Encircled/white A, B and C show monomers in high-level of theory. Cyan A, B and C show the embedded monomers.

The subtractive version of Equation S5 is Equation S12.

$$\begin{aligned}\Xi_{\text{y}} =& \Xi^{\text{low}} + \sum^{\text{trimer}} E_{\text{dimers}}^{\text{embedded,high}} - \sum^{\text{trimer}} E_{\text{dimers}}^{\text{embedded,low}} \\ &- \sum^{\text{trimer}} E_{\text{monomers}}^{\text{embedded-monomers,high}} + \sum^{\text{trimer}} E_{\text{monomers}}^{\text{embedded-monomers,low}}\end{aligned} \tag{S12}$$

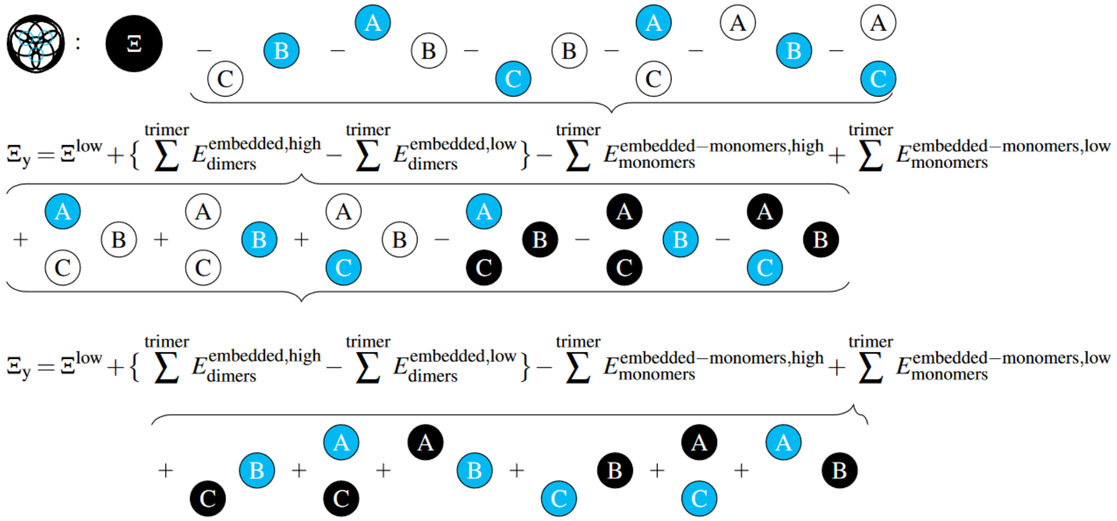

Figure S16: Schematic of Equation S12. Black A, B and C represent different monomers at low-level of theory. Encircled/white A, B and C show monomers in high-level of theory. Cyan A, B and C show the embedded monomers.

In the unembedded case, we were able to see an increase in accuracy by excluding the subsystem energies for subtractive approaches and only take the interaction energies into account. Applying the same ansatz at the embedded schemes results in Equation S13.

$$\begin{aligned}\Xi_{\text{odww}} =& \Xi^{\text{low}} + \sum^{\text{trimer}} E_{\text{dimers}}^{\text{embedded,high}} - \sum^{\text{trimer}} E_{\text{dimers}}^{\text{embedded,low}} \\ &- 2 \sum^{\text{trimer}} E_{\text{monomers}}^{\text{high}} + 2 \sum^{\text{trimer}} E_{\text{monomers}}^{\text{low}}\end{aligned} \tag{S13}$$

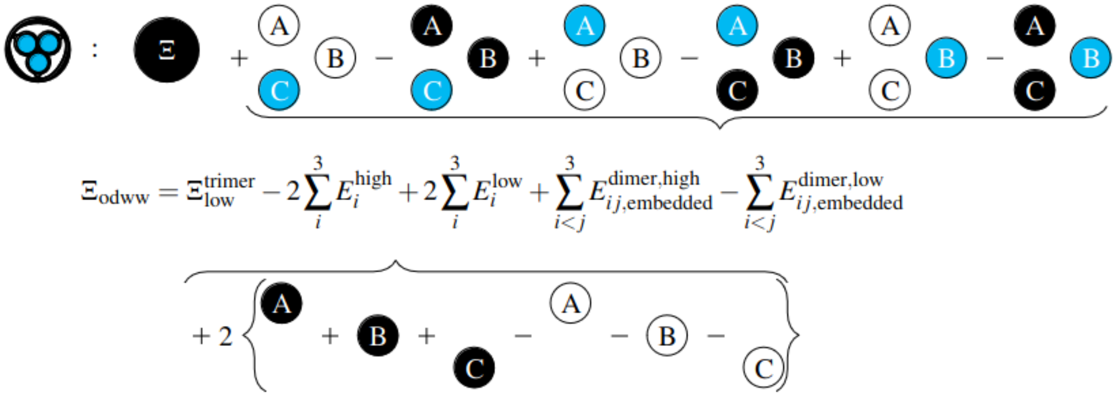

Figure S17: example Schematic of Equation S13 with monomers as sub-systems. Black A, B and C represent different monomers at low-level of theory. Encircled/white A, B and C show monomers in high-level of theory. Cyan A, B and C show the embedded monomers.

The following scheme, Equation S14, results most of the time in low errors and is therefore recommended. The highest amount of information generated from embedding in a trimer comes from the dimer in monomer embedding, and it comes as no surprise that this term needs to be included. The subtraction of monomer in dimer embedded calculations removes parts of the trimer interaction energy that are otherwise double counted. Finally, removing the existing energy difference from high- to low-level of theory on the monomer level only leaves the low-level trimer interaction with the embedded interaction energy differences for three- and two-body interaction.

$$\begin{aligned}\Xi_z =& \Xi^{\text{low}} + \sum^{\text{trimer}} E^{\text{embedded,high}}_{\text{dimers}} - \sum^{\text{trimer}} E^{\text{embedded,low}}_{\text{dimers}} \\ &- \sum^{\text{trimer}} E^{\text{embedded}-\text{dimers,high}}_{\text{monomers}} + \sum^{\text{trimer}} E^{\text{embedded}-\text{dimers,low}}_{\text{monomers}} \\ &- \sum^{\text{trimer}} E^{\text{high}}_{\text{monomers}} + \sum^{\text{trimer}} E^{\text{low}}_{\text{monomers}}\end{aligned} \tag{S14}$$

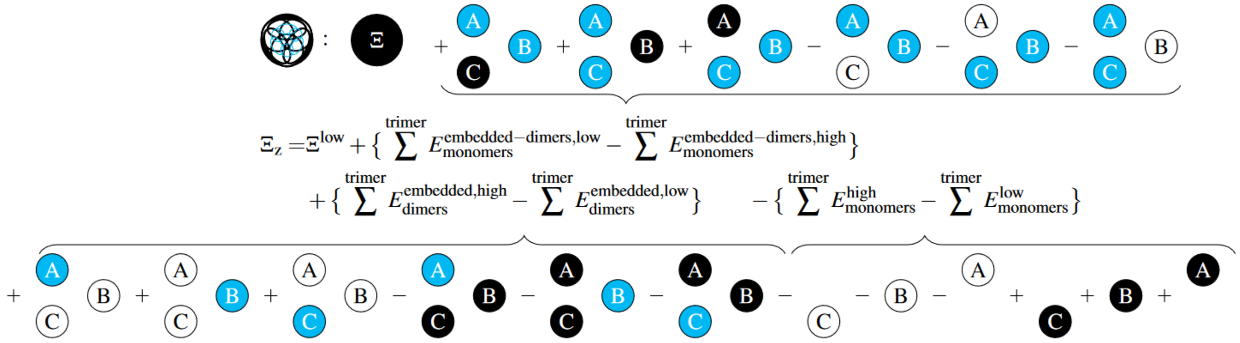

Figure S18: Schematic of Equation S12. Black A, B and C represent different monomers at low-level of theory. Encircled/white A, B and C show monomers in high-level of theory. Cyan A, B and C show the embedded monomers.

If we take Equation S14 and do not remove the monomer energy difference between high- and low-level of theories, we need to subtract once more monomer energies to obtain a single trimer, given in Equation S15.

$$\begin{aligned} \Xi_q =& \Xi^{low} + \sum^{trimer} E^{embedded,high}_{dimers} - \sum^{trimer} E^{embedded,low}_{dimers} \\ &+ 2 \sum^{trimer} E^{embedded-dimers,high}_{monomers} - 2 \sum^{trimer} E^{embedded-dimers,low}_{monomers} \end{aligned} \tag{S15}$$

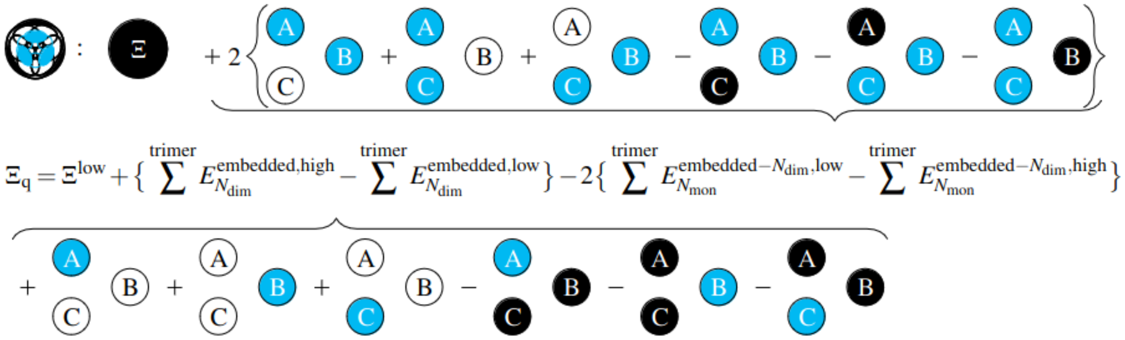

Figure S19: Schematic of Equation S15. Black A, B and C represent different monomers at low-level of theory. Encircled/white A, B and C show monomers in high-level of theory. Cyan A, B and C show the embedded monomers.

Now we understand the various schemes and get finally see the best performances for each method applied. The following figures are the full versions of Figure 3, Figure 5,Figure 6 and Figure 4 from the main text.

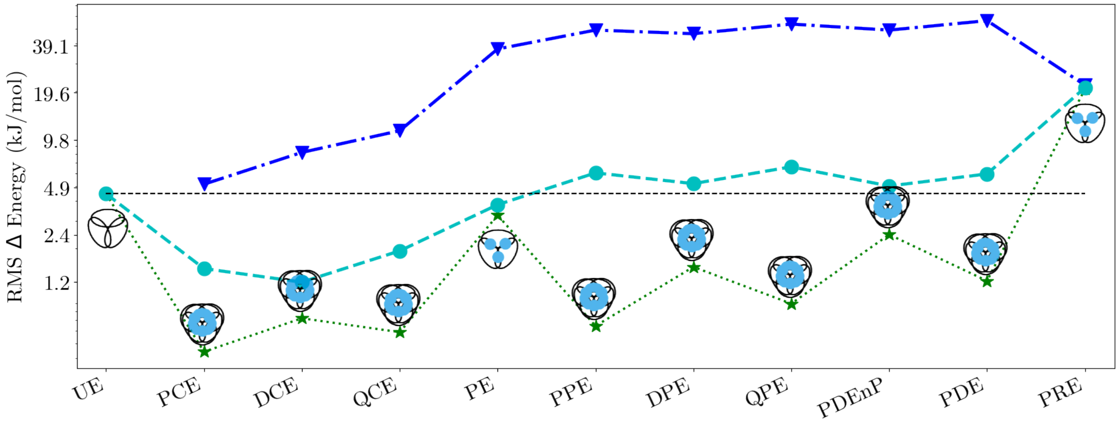

(a)

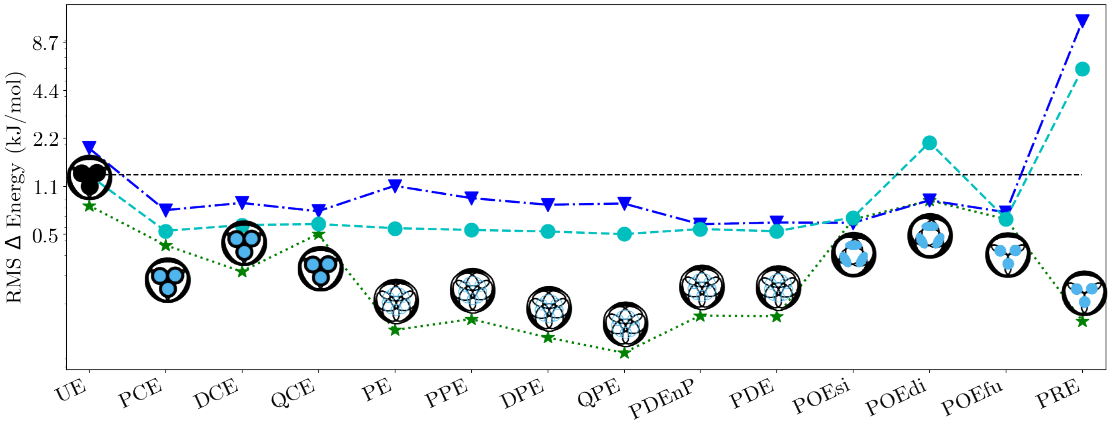

(b)

Figure S20: Additive schemes (a) and subtractive schemes (b) for all trimer systems for each embedding method. Both plots are further separated into monomer (Ξ_{pmon} Equation S2/ Ξ_{omp} Equation S8) and dimer (Ξ_{pdim} Equation 3 or Ξ_{d} Equation S4/ Ξ_{odp} Equation S9) approximations as well as the corresponding *best* schemes, see Table S22 with corresponding symbol for each point. The *best* scheme is shown in green, the dimer approximations (exact two-body interaction) in cyan, and the monomer approximations in blue. All root-mean-squared (RMS) errors are on a logarithmic scale. The dashed line represents the result from the unembedded method. This figure is the full version of Figure 3.

All additive scheme in the comparison of all trimers yields rather large errors, around 4.81 kJ/mol on the basic dimer level. This is reduced to 0.71 kJ/mol when using point charges with equation Equation S7 in section S2, rather than the standard scheme. However, as it is also the case for the standard Ξ_{pdim} scheme, going beyond point charge embedding does

not yield huge benefits in this respect.

As could be seen with the additive approaches, a more sophisticated scheme, Equation S1, will reduce the error further to 1.13 kJ/mol. Whereas point charge embedding reduced the error to 0.92 kJ/mol for the standard scheme Equation S4, there is no improvement for either including dipoles, quadrupoles, and polarizabilites. However, the combination of dipole and polarizability embedding is finally reducing the error to as little as 0.28 kJ/mol. For our standard scheme, also being the best scheme for this method, this also yields the lowest error over all trimers, thus going beyond the error of the unembedded subtractive scheme by about a factor of six. This result is only surpassed by employing projection-based embedding, able to reduce the error to 0.10 kJ/mol using Equation S10.

Projection-based density embedding performed in this study well and is recommended in regards to accuracy. However, the downside is that its computational time is approximately ten times higher than the unembedded method. Surprisingly for us, taking account for only electrostatics and polarisable-terms already gives exceptionally low errors, while keeping the computational costs rather low, this would be recommenced for a fast but still accurate approach.

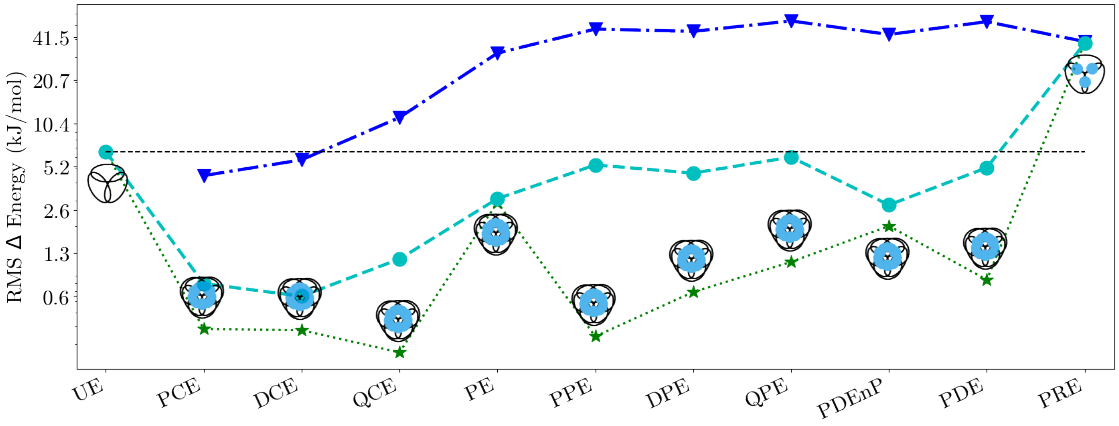

(a)

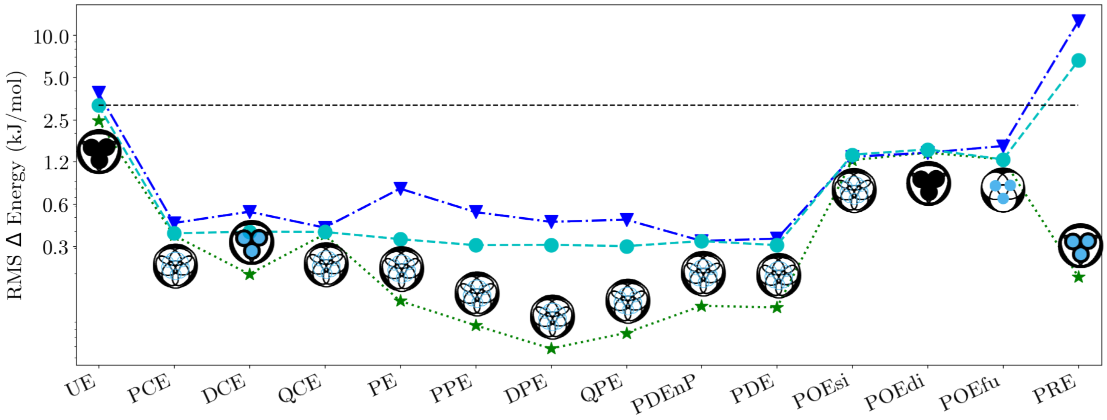

(b)

Figure S21: a:Additive schemes for induction dominated trimer systems each embedding method. b: Subtractive schemes for induction dominated trimer systems each embedding method. Both further separated into monomer and dimer approximations as well as the corresponding best scheme (see Table S25 with corresponding symbol for each point, The *best* scheme is shown in green, the dimer approximations in cyan, and the monomer approximations in blue. All root-mean-squared (RMS) errors are on a logarithmic scale. The dashed line represents the result from the unembedded method. This figure is the full version of Figure 4.

Using the best subtractive scheme yields an improvement when going from point charges to dipole moments. In addition, when including polarizable effects and then subsequently point charges, etc. systematically improves the error.

Nevertheless, the errors in a subtractive scheme can be reduced for these induction dominated trimers from 2.46 kJ/mol down to 0.06 kJ/mol when using dipoles and polarizable

terms and down to 0.19 kJ/mol for the projection-based density embedding, which is more than one order of magnitude.

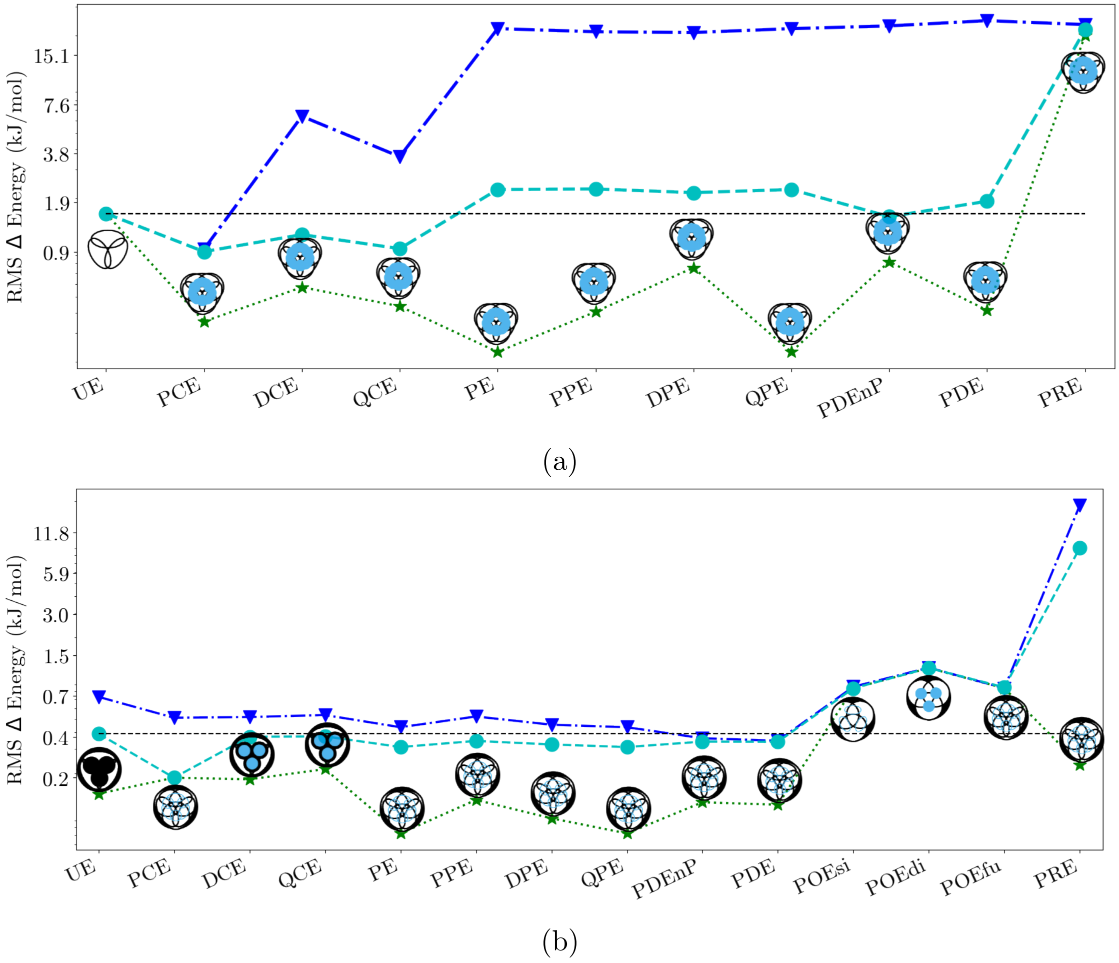

Figure S22: Additive schemes (a) and subtractive schemes (b) for the dispersion group defined via Equation 14 trimer systems for each embedding method. Both plots are further separated into monomer (Ξ_{pmon} Equation S2/ Ξ_{omp} Equation S8) and dimer (Ξ_{pdim} Equation 3 or Ξ_{d} Equation S4/ Ξ_{odp} Equation S9) approximations as well as the corresponding *best* schemes, see Table S22 with corresponding symbol for each point. The *best* scheme is shown in green, the dimer approximations in cyan, and the monomer approximations in blue. All root-mean-squared (RMS) errors are on a logarithmic scale. The dashed line represents the result from the unembedded method. this figure is the full version of Figure 5.

For the additive schemes, the error is reduced by the point charge embedding if equation Equation S7. However, from this on, the error behaves rather erratically, as dipole and

quadrupole embedding yield a higher error than the point charges, polarizable embedding will yield again a lower error, with point charged and dipoles added to it yielding worse and quadrupole polarizable embedding yielding lower errors.

A similar behaviour can be observed for the best subtractive schemes in combination with the different methods.

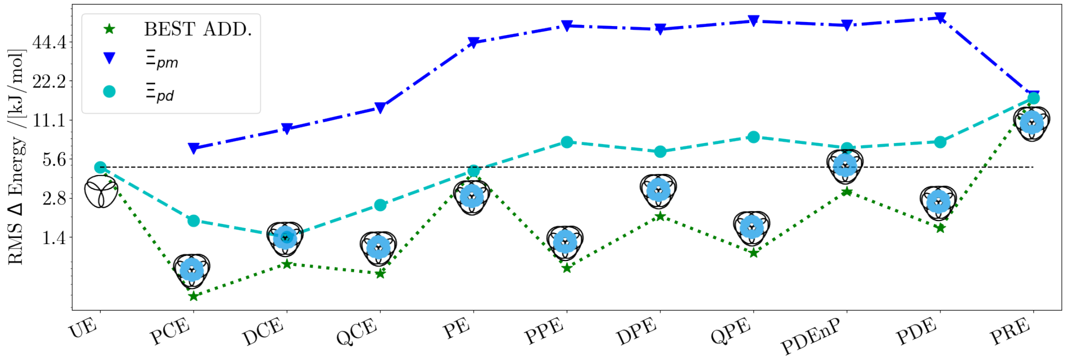

(a)

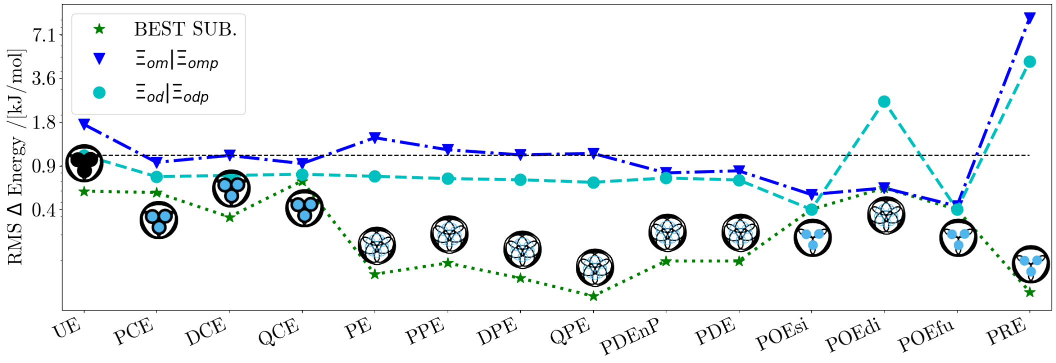

(b)

Figure S23: (a)Additive schemes for mixed interaction trimer systems each embedding method. (b) Subtractive schemes for mixed interaction trimer systems each embedding method. Both further separated into monomer and dimer approximations as well as the corresponding best scheme (see Table S24 with corresponding symbol for each point, Green=Best Scheme, Cyan=Dimer Equation 3 and Equation S4 respectively, Blue=Monomer calculations, Equation S2. All RMS errors are on a logarithmic scale. The dashed line represents the result from the unembedded method. this figure is the full version of Figure 6.

For the best additive scheme, the point charge embedding yields a considerable improvement of a factor of nine compared to the unembedded method.
Still, adding dipole moments or quadrupole moments does not improve the results, as well as adding polarizable effects or density embedding. For the subtractive scheme, however, the results are considerably better when including any polarizable terms into the mix: Here, the error is reduced for the best subtractive schemes from 0.60 kJ/mol (unembedded) to 0.16 kJ/mol for the polarizable embedding. Density embedding also gives some improvement, with the best method being the projection-based density embedding with an error of 0.12 kJ/mol.
Table S22 to Table S25 give an overview which schemes worked best for which method for each of the embedding methods. Ξ_{d}, Equation S14 and Ξ_{pd3}, Equation S7 performed most of the time quite well.

S3 Set of Molecules

In this section, we further define the investigated systems. The systems which were taken out of the 3B-69 benchmarkset are: 1a-1c,3a-3c,6a-6c,7a-7c,9a-9c,10a-10c,12a-12c,14a-14c,20a-20c, as well as two additional trimers. These were "trimer H", another water trimer, and "Trimer N", an ammonia trimer. To increase the number of potentially H-bonded system and to have an ammonia trimer as a bridge to another benchmark set (X23revised[1]). The selected trimers from 3B-69 were chosen due to their high trimer interaction energy.[2] The structures of our trimers are listed below in xyz format.

12

Trimer N

N 0.7293907555 1.6924589234 0.0000626978

H	1.1406566402	0.7592540074	-0.0003931395
H	1.091137294	2.1789758975	-0.8108802174
H	1.0931660859	2.1791668581	0.8099794227
N	-1.8304068651	-0.2145170313	-0.0002004591
H	-1.228963655	0.6090487896	-0.0000866107
H	-2.4357098032	-0.1426928132	-0.8086750555
H	-2.430891845	-0.1462590946	0.8121746524
N	1.1010573641	-1.4778193076	-0.0003247397
H	0.0871619586	-1.3682189549	-0.0023644575
H	1.3430642421	-2.0374223266	-0.8086895494
H	1.3400903012	-2.0327104547	0.8121724621

9

Trimer H

H	2.554686	2.938700	2.595663
H	4.036937	3.241557	2.539937
O	3.280386	3.058020	1.983002
H	0.589974	1.477270	3.891526
H	1.106449	1.562453	2.471000
O	1.048524	2.060286	3.286500
H	2.480998	1.668301	1.046915
H	1.541061	1.219095	-0.051551
O	1.801010	1.024388	0.848863

The obtained R_{ID} values for each trimer system are listed in Table S3 below.

Table S3: R_{ID} values for each system

System	R_{ID}	Category	System	R_{ID}	Category
1a	0.69	Induction	10a	0.38	Mixed
1b	0.68	Induction	10b	0.11	Dispersion
1c	0.55	Mixed	10c	0.74	Induction
3a	0.62	Mixed	12a	0.49	Mixed
3b	0.46	Mixed	12b	0.05	Dispersion
3c	0.50	Mixed	12c	0.29	Dispersion
6a	0.57	Mixed	14a	0.25	Dispersion
6b	0.76	Induction	14b	0.44	Mixed
6c	0.46	Mixed	14c	0.31	Dispersion
7a	0.12	Dispersion	20a	0.49	Mixed
7b	0.59	Mixed	20b	0.90	Induction
7c	0.58	Mixed	20c	0.37	Mixed
9a	0.43	Mixed	NH_3	0.36	Mixed
9b	0.48	Mixed	H_2O	0.54	Mixed
9c	0.46	Mixed			

S3.1 DALTON

In this subsection, we give a brief overview of the analysis done for PE-DALTON calculations in the Ξ_{pd3} scheme. Including embedding energy contributions to the obtained energies results in double counting, therefore lowering the accuracy. This Analysis was done for all schemes and all DALTON methods. Although taking only electronic (elec.) and nuclear (nuc.) (E_EN, "embedding, elec. and nuc.") contributions into account, this does not always result in the best outcome (due to error cancellation). It is still the most reasonable and stable approach and therefore recommended. The core region response is included and double counting becomes impossible.

Table S4: Errors for various energy terms of the DALTON polarizable density embedding scheme Ξ_{pmon} in kJ/mol. The label "E_" stands for embedding, "_E" stands for electronic, "_N" for nuclear, "_R" for repulsion, and "_P" for polarised. The columns "onlypol", "nopol" and "nopol+pol" stand for only considering the polarised contribution, only consider the not polarised contribution and considering both, respectively.

	RMS Errors			Maximal Errors		
Energy contribution	onlypol	nopol	nopol+pol	onlypol	nopol	nopol+pol
only elec. E_E	137.29	677.16	699.32	412.77	2080.96	1905.01
only nuc. E_N	236.49	697.14	719.84	475.59	2066.24	1894.27
elec. nuc. E_EN	65.49	21.12	44.32	115.53	40.50	80.32
elec. rep. E_ER	137.29	665.92	675.39	412.77	2116.39	1940.45
nuc. rep. E_NR	236.49	721.98	18.91	475.59	2030.80	47.81
pol. E_P	59.66	6.24	59.66	104.34	173.216	104.34
elec.+pol. E_EP	150.09	677.16	711.93	384.85	2080.96	1898.72
nuc.+pol. E_NP	211.89	697.14	706.74	416.95	2066.24	1900.57
rep. E_R	6.236	160.54	160.54	39.324	291.23	291.23
pol.+rep. E_PR	59.66	160.54	130.90	104.34	291.23	222.35
elec.+nuc.+rep. E_ENR	65.49	53.06	31.11	115.53	95.38	49.64
nuc.+pol.+rep. E_NPR	211.89	721.98	738.22	416.95	2030.80	1865.14
elec.+pol.+rep. E_EPR	150.09	665.92	685.36	384.85	2116.39	1934.15
elec.+nuc.+pol. E_ENP	36.77	21.12	74.02	61.53	40.496	149.19
only EN E.N.	6.236	89.316		39.324	173.216	

Table S5: Errors for various energy terms of the DALTON polarizable density embedding scheme Ξ_{pm2} in kJ/mol. The label "_E" stands for electronic, "_N" for nuclear, "_R" for repulsion, and "_P" for polarised. The columns "onlypol", "nopol" and "nopol+pol" stand for only considering the polarised contribution, only consider the not polarised contribution and considering both, respectively.

	RMS Errors			Maximal Errors		
Energy contribution	onlypol	nopol	nopol+pol	onlypol	nopol	nopol+pol
only elec. E_E	60.77	447.58	453.16	173.33	1390.39	1313.71
only nuc. E_N	151.92	470.39	482.12	304.69	1375.11	1300.64
elec. nuc. E_EN	62.41	9.60	11.39	110.25	19.32	24.55
elec. rep. E_ER	60.77	442.05	439.94	173.33	1414.43	1337.75
nuc. rep. E_NR	62.41	488.20	505.08	304.69	1351.07	1276.60
pol. E_P	66.06	75.94	66.06	121.10	144.06	121.10
% elec.+pol. E_EP	62.03	447.58	456.73	164.03	1390.39	1311.62
nuc.+pol. E_NP	142.83	470.39	477.55	285.14	1375.11	1302.74
rep. E_R	30.48	123.12	123.14	44.29	222.07	222.07
pol.+rep. E_PR	66.06	123.12	113.25	121.10	222.07	199.11
elec.+nuc.+rep. E_ENR	62.41	53.26	39.94	110.25	96.16	62.36
nuc.+pol.+rep. E_NPR	142.83	488.20	499.80	285.14	1351.07	1278.70
elec.+pol.+rep. E_EPR	62.03	442.05	442.56	164.03	1414.43	1335.65
elec.+nuc.+pol. E_ENP	52.66	9.60	19.93	87.30	19.32	38.61

Table S6: Errors for various energy terms of the DALTON polarizable density embedding scheme Ξ_{pdim} in kJ/mol. The label "_E" stands for electronic, "_N" for nuclear, "_R" for repulsion, and "_P" for polarised. The columns "onlypol", "nopol" and "nopol+pol" stand for only considering the polarised contribution, only consider the not polarised contribution and considering both, respectively.

	RMS Errors			Maximal Errors		
Energy contribution	onlypol	nopol	nopol+pol	onlypol	nopol	nopol+pol
only elec. E_E	97.48	2.25	103.91	350.26	5.80	364.60
only nuc. E_N	102.18	9.30	102.18	357.69	20.80	357.69
elec. nuc. E_EN	1.03	2.25	10.86	2.62	5.80	23.94
elec. rep. E_ER	97.48	3.05	102.76	350.26	8.11	358.41
nuc. rep. E_NR	102.18	9.69	103.42	357.69	19.01	363.89
pol. E_P	11.27	11.27	11.27	30.53	30.53	30.53
elec.+pol. E_EP	97.48	2.25	103.91	350.26	5.80	364.60
nuc.+pol. E_NP	102.18	9.30	102.18	357.69	20.80	357.69
rep. E_R	11.27	9.69	9.69	30.53	19.01	19.01
pol.+rep. E_PR	11.27	9.69	9.69	30.53	19.01	19.01
elec.+nuc.+rep. E_ENR	1.03	3.05	11.27	2.62	8.11	30.53
nuc.+pol.+rep. E_NPR	102.18	9.69	103.42	357.69	19.01	363.89
elec.+pol.+rep. E_EPR	97.48	3.05	102.76R	350.26	8.11	358.41
elec.+nuc.+pol. E_ENP	1.03	2.25	10.86	2.62	5.80	23.94

Table S7: Errors for various energy terms of the DALTON polarizable density embedding scheme Ξ_{pd2} in kJ/mol. The label "_E" stands for electronic, "_N" for nuclear, "_R" for repulsion, and "_P" for polarised. The columns "onlypol", "nopol" and "nopol+pol" stand for only considering the polarised contribution, only consider the not polarised contribution and considering both, respectively.

	RMS Errors			Maximal Errors		
Energy contribution	onlypol	nopol	nopol+pol	onlypol	nopol	nopol+pol
only elec. E_E	63.34	451.34	458.27	158.09	1387.49	1317.97
only nuc. E_N	132.89	464.33	471.97	271.96	1378.12	1310.80
elec. nuc. E_EN	44.43	13.67	26.65	77.74	25.82	48.20
elec. rep. E_ER	63.34	444.10	443.91	158.09	1410.87	1341.35
nuc. rep. E_NR	132.89	480.31	493.01	271.96	1354.75	1287.42
pol. E_P	21.90	21.90	21.90	29.73	29.73	29.73
elec.+pol. E_EP	63.34	451.34	458.27	158.09	1387.49	1317.97
nuc.+pol. E_NP	132.89	464.33	471.97	271.96	1378.12	1310.79
rep. E_R	21.90	104.63	104.63	29.73	189.19	189.19
pol.+rep. E_PR	21.90	104.63	104.63	29.73	189.19	189.19
elec.+nuc.+rep. E_ENR	44.43	34.85	21.90	77.74	63.25	29.73
nuc.+pol.+rep. E_NPR	132.89	480.31	493.01	271.96	1354.75	1287.42
elec.+pol.+rep. E_EPR	63.34	444.10	443.91	158.09	1410.87	1341.35
elec.+nuc.+pol. E_ENP	44.43	13.67	26.65	77.74	25.82	48.20

Table S8: Errors for various energy terms of the DALTON polarizable density embedding scheme Ξ_{pd3} in kJ/mol. The label "_E" stands for electronic, "_N" for nuclear, "_R" for repulsion, and "_P" for polarised. The columns "onlypol", "nopol" and "nopol+pol" stand for only considering the polarised contribution, only consider the not polarised contribution and considering both, respectively.

	RMS Errors			Maximal Errors		
Energy contribution	onlypol	nopol	nopol+pol	onlypol	nopol	nopol+pol
only elec. E_E	18.36	1.21	18.05	43.70	2.92	43.78
only nuc. E_N	16.98	1.88	16.98	44.69	4.39	44.69
elec. nuc. E_EN	1.81	1.21	1.31	4.14	2.92	3.21
elec. rep. E_ER	18.36	2.15	16.19	43.70	4.09	40.66
nuc. rep. E_NR	16.98	2.43	18.91	44.69	8.20	47.81
pol. E_P	31.37	1.88	31.37	69.06	4.39	69.06
elec E_EP	43.02	711.93	42.58	75.32	1898.72	74.21
nuc.+pol. E_NP	27.28	1.88	27.28	72.32	4.39	72.32
rep. E_R	29.09	2.43	2.43	69.55	8.20	8.20
pol.+rep. E_PR	31.37	2.43	29.40	69.06	8.20	68.80
elec.+nuc.+rep. E_ENR	1.81	2.15	2.23	4.14	4.09	4.01
nuc.+pol.+rep. E_NPR	27.29	2.43	26.30	72.32	8.20	72.07
elec.+pol.+rep. E_EPR	43.02	2.15	40.35	75.32	4.09	72.77
elec.+nuc.+pol. E_ENP	31.51	1.21	31.07	69.90	2.92	69.80

Table S9: Errors for various energy terms of the DALTON polarizable density embedding scheme Ξ_{pmon} in kJ/mol. The label "_E" stands for electronic, "_N" for nuclear, "_R" for repulsion, and "_P" for polarised. The columns "onlypol", "nopol" and "nopol+pol" stand for only considering the polarised contribution, only consider the not polarised contribution and considering both, respectively.

	RMS Errors			Maximal Errors		
Energy contribution	onlypol	nopol	nopol+pol	onlypol	nopol	nopol+pol
only elec.	184.04	692.10	758.46	395.06	2090.42	1878.62
only nuc.	299.21	716.07	784.10	635.69	2079.27	1871.97
elec. nuc.	57.76	14.19	41.44	109.75	32.46	81.16
elec. rep.	184.04	692.10	758.46	395.06	2090.42	1878.62
nuc. rep.	299.21	716.07	38.42	635.69	2079.27	110.38
multipole	43.01	47.66	47.41	70.92	160.72	98.03
elec.+multipole	217.79	692.10	781.62	494.14	2090.42	1872.70
nuc.+multipole	259.70	716.07	761.15	536.61	2079.27	1877.89
multipole+rep.	43.01	86.23	43.01	70.92	176.66	70.92
elec.+nuc.+rep.	57.76	14.19	41.44	109.75	32.46	81.16
nuc.+multipole+rep.	259.70	716.07	761.15	536.61	2079.27	1877.89
elec.+multipole+rep.	217.79	692.10	781.62	494.14	2090.42	1872.70
elec.+nuc.+multipole	20.10	14.19	86.65	49.27	32.46	183.70

Table S10: Errors for various energy terms of the DALTON polarizable density embedding scheme Ξ_{pm2} in kJ/mol. The label "_E" stands for electronic, "_N" for nuclear, "_R" for repulsion, and "_P" for polarised. The columns "onlypol", "nopol" and "nopol+pol" stand for only considering the polarised contribution, only consider the not polarised contribution and considering both, respectively.

	RMS Errors			Maximal Errors		
Energy contribution	onlypol	nopol	nopol+pol	onlypol	nopol	nopol+pol
only elec.	83.23	456.90	489.35	193.09	1396.86	1295.33
only nuc.	195.50	483.41	525.48	403.27	1383.68	1284.63
elec. nuc.	57.01	12.29	9.50	105.93	27.82	21.05
elec. rep.	83.23	456.90	489.35	193.09	1396.86	1295.33
nuc. rep.	195.50	483.41	525.48	403.27	1383.68	1284.63
multipole	58.28	54.25	40.54	108.17	139.50	102.86
elec.+multipole	94.74	456.90	496.33	226.12	1396.86	1293.36
nuc.+multipole	181.20	483.41	517.46	370.25	1383.68	1286.61
multipole+rep.	58.28	72.90	58.28	108.17	144.81	108.17
elec.+nuc.+rep.	57.01	12.29	9.50	105.93	27.82	21.05
nuc.+multipole+rep.	181.20	483.41	517.46	370.25	1383.68	1286.61
elec.+multipole+rep.	94.74	456.90	496.33	8.00	1396.86	1293.36
elec.+nuc.+multipole	42.79	12.29	23.03	69.29	27.82	51.038

Table S11: Errors for various energy terms of the DALTON polarizable density embedding scheme Ξ_{pdim} in kJ/mol. The label "_E" stands for electronic, "_N" for nuclear, "_R" for repulsion, and "_P" for polarised. The columns "onlypol", "nopol" and "nopol+pol" stand for only considering the polarised contribution, only consider the not polarised contribution and considering both, respectively.

	RMS Errors			Maximal Errors		
Energy contribution	onlypol	nopol	nopol+pol	onlypol	nopol	nopol+pol
only elec.	97.47	1.81	104.62	342.36	4.38	357.81
only nuc.	103.93	10.45	103.93	356.53	20.70	356.53
elec. nuc.	1.05	1.81	12.05	3.27	4.38	28.36
elec. rep.	97.47	1.81	104.62	342.36	4.38	357.81
nuc. rep.	103.93	10.45	103.93	356.53	20.70	356.53
multipole	12.05	12.05	12.05	28.36	28.36	28.36
elec.+multipole	97.47	1.81	104.62	342.36	4.38	357.81
nuc.+multipole	103.93	10.45	103.93	356.53	20.70	356.53
multipole+rep.	10.45	10.45	10.45	20.70	20.70	20.70
elec.+nuc.+rep.	1.05	1.81	12.05	3.27	4.38	28.36
nuc.+multipole+rep.	103.93	10.45	103.93	356.53	20.70	356.53
elec.+multipole+rep.	97.47	1.81	104.62	342.36	4.38	357.81
elec.+nuc.+multipole	1.05	1.81	12.05	3.27	4.38	28.36

Table S12: Errors for various energy terms of the DALTON polarizable density embedding scheme Ξ_{pd2} in kJ/mol. The label "_E" stands for electronic, "_N" for nuclear, "_R" for repulsion, and "_P" for polarised. The columns "onlypol", "nopol" and "nopol+pol" stand for only considering the polarised contribution, only consider the not polarised contribution and considering both, respectively.

	RMS Errors			Maximal Errors		
Energy contribution	onlypol	nopol	nopol+pol	onlypol	nopol	nopol+pol
only elec.	97.00	461.55	497.35	207.46	1393.59	1289.42
only nuc.	179.57	476.44	514.18	358.35	1386.37	1284.77
elec. nuc.	38.59	10.02	24.74	73.16	22.59	46.17
elec. rep.	97.00	461.55	497.35	207.46	1393.59	1289.42
nuc. rep.	179.57	476.44	514.18	358.35	1386.37	1284.77
multipole	24.74	24.74	24.74	46.17	46.17	46.17
elec.+multipole	97.00	461.55	497.35	207.46	1393.59	1289.42
nuc.+multipole	179.57	476.44	514.18	358.35	1386.37	1284.77
multipole+rep.	54.21	54.21	54.21	111.33	111.33	111.33
elec.+nuc.+rep.	38.59	10.02	24.74	73.16	22.59	46.17
nuc.+multipole+rep.	179.57	476.44	514.18	358.35	1386.37	1284.77
elec.+multipole+rep.	97.00	461.55	497.35	207.46	1393.59	1289.42
elec.+nuc.+multipole	38.59	10.02	24.74	73.16	22.59	46.17

Table S13: Errors for various energy terms of the DALTON polarizable density embedding scheme Ξ_{pd3} in kJ/mol. The label "_E" stands for electronic, "_N" for nuclear, "_R" for repulsion, and "_P" for polarised. The columns "onlypol", "nopol" and "nopol+pol" stand for only considering the polarised contribution, only consider the not polarised contribution and considering both, respectively.

	RMS Errors			Maximal Errors		
Energy contribution	onlypol	nopol	nopol+pol	onlypol	nopol	nopol+pol
only elec.	38.31	1.33	38.97	112.15	2.52	112.95
only nuc.	38.42	1.55	38.42	110.38	4.41	110.38
elec. nuc.	0.61	1.33	1.21	1.34	2.52	2.85
elec. rep.	38.31	1.33	38.97	112.15	2.52	112.95
nuc. rep.	38.42	1.55	38.42	110.38	4.41	110.38
multipole	44.69	72.28	113.05	108.05	128.87	199.22
elec.+multipole	54.54	781.62	55.05	142.45	1872.70	144.96
nuc.+multipole	63.71	1.55	63.71	156.47	4.41	156.47
multipole+rep.	44.69	1.55	44.69	108.05	4.41	108.06
elec.+nuc.+rep.	0.61	1.33	1.21	1.34	2.52	2.85
nuc.+multipole+rep.	63.71	1.55	63.71	156.47	4.41	156.47
elec.+multipole+rep.	54.54	1.33	55.05	142.45	2.52	144.96
elec.+nuc.+multipole	45.77	1.33	45.84	110.18	2.52	111.32

Table S14: Errors for various energy terms of the DALTON density embedding scheme Ξ_{pmon} in kJ/mol. The label "elec" stands for electronic, "nuc" for nuclear, "rep" for repulsion.

Energy contribution	RMS Errors	Maximal Errors
only elec.	677.38	2081.19
only nuc.	693.39	2066.66
elec. nuc.	23.12	51.37
elec. rep.	666.27	2116.20
nuc. rep.	716.04	2031.65
rep.	213.98	362.15
elec.+nuc.+rep.	49.76	85.81

Table S15: Errors for various energy terms of the DALTON density embedding scheme Ξ_{pm2} in kJ/mol. The label "elec" stands for electronic, "nuc" for nuclear, "rep" for repulsion.

Energy contribution	RMS Errors	Maximal Errors
only elec.	447.68	1390.50
only nuc.	468.21	1375.40
elec. nuc.	9.88	19.33
elec. rep.	442.10	1414.34
nuc. rep.	484.83	1351.56
rep.	114.44	198.67
elec.+nuc.+rep.	51.54	90.96

Table S16: Errors for various energy terms of the DALTON density embedding scheme Ξ_{pdim} in kJ/mol. The label "elec" stands for electronic, "nuc" for nuclear, "rep" for repulsion.

Energy contribution	RMS Errors	Maximal Errors
only elec.	39.94	214.76
only nuc.	49.65	265.51
elec. nuc.	14.22	75.68
elec. rep.	56.02	301.01
nuc. rep.	33.80	179.26
rep.	140.35	231.07
elec.+nuc.+rep.	4.15	10.58

Table S17: Errors for various energy terms of the DALTON density embedding scheme Ξ_{pd2} in kJ/mol. The label "elec" stands for electronic, "nuc" for nuclear, "rep" for repulsion.

Energy contribution	RMS Errors	Maximal Errors
only elec.	451.89	1387.59
only nuc.	463.16	1378.44
elec. nuc.	13.48	25.51
elec. rep.	444.69	1410.80
nuc. rep.	478.06	1355.23
rep.	96.50	167.22
elec.+nuc.+rep.	33.28	58.39

Table S18: Errors for various energy terms of the DALTON density embedding scheme Ξ_{pd3} in kJ/mol. The label "elec" stands for electronic, "nuc" for nuclear, "rep" for repulsion.

Energy contribution	RMS Errors	Maximal Errors
only elec.	48.45	260.79
only nuc.	48.13	258.41
elec. nuc.	1.40	3.08
elec. rep.	48.97	263.51
nuc. rep.	47.62	255.69
elec.+pol.	48.45	260.79
nuc.+pol.	48.13	258.41
rep.	3.75	9.47
elec.+nuc.+rep.	2.03	4.18

Table S19: Errors for various schemes of the DALTON quadrupole embedding method in kJ/mol.

**QP**	RMS Errors	Maximal Errors
Ξ_{pdim} noemb	4.96	10.25
Ξ_{pmon}	18.18	33.98
Ξ_{pm2}	10.35	20.73
Ξ_{pdim}	2.90	6.90
Ξ_{pd2}	11.81	23.11
Ξ_{pd3}	0.89	2.01

Table S20: Errors for various schemes of the DALTON dipole embedding method in kJ/mol.

**DP**	RMS Errors	Maximal Errors
Ξ_{pdim} noemb	4.96	10.25
Ξ_{pmon}	12.76	29.46
Ξ_{pm2}	16.80	32.12
Ξ_{pdim}	1.95	4.29
Ξ_{pd2}	8.64	20.45
Ξ_{pd3}	1.10	1.97

Table S21: Errors for various schemes of the DALTON point charge embedding method in kJ/mol.

**PC**	RMS Errors	Maximal Errors
Ξ_{pdim} noemb	4.96	10.25
Ξ_{pmon}	8.11	24.54
Ξ_{pm2}	18.41	37.35
Ξ_{pdim}	2.24	6.34
Ξ_{pd2}	5.36	14.57
Ξ_{pd3}	0.71	1.42

S4 RMS Tables

In this section, the root mean square errors for various schemes, groups, and different methods are provided in detail.

Table S22: Errors for basic schemes and best schemes in additive and subractive/ONIOM category for each method for all trimer systems in kJ/mol.

RMS, all trimers	unembedded	PCE	DCE	QCE
Ξ_{omp}	1.89	0.77	0.85	0.76
Ξ_{odp}	1.28	0.57	0.62	0.63
Best ONIOM scheme	0.82	0.46	0.32	0.55
	Ξ_{odw}	Ξ_{odww}	Ξ_{odww}	Ξ_{odww}
Ξ_{pmon}	-	5.12	8.16	11.22
Ξ_{pdim}	4.46	1.49	1.22	1.93
Best monomer scheme	-	5.12	8.16	11.22
	-	Ξ_{pmon}	Ξ_{pmon}	Ξ_{pm2}
Best dimer scheme	4.46	0.44	0.72	0.59
	Ξ_{pdim}	Ξ_{pd3}	Ξ_{pd3}	Ξ_{pd3}

RMS, all trimer	PE	PPE	DPE	QPE
Ξ_{omp}	1.09	0.92	0.83	0.85
Ξ_{odp}	0.59	0.58	0.57	0.54
Best ONIOM scheme	0.14	0.16	0.12	0.10
	Ξ_{z}	Ξ_{z}	Ξ_{z}	Ξ_{z}
Ξ_{pmon}	37.11	48.93	46.37	53.44
Ξ_{pdim}	3.780	6.05	5.17	6.61
Best monomer scheme	36.90	47.01	45.16	53.44
	Ξ_{pm2}	Ξ_{pm2}	Ξ_{pm2}	Ξ_{pmon}
Best dimer scheme	3.26	0.64	1.53	0.89
	Ξ_{pdim}	Ξ_{pd3}	Ξ_{pd3}	Ξ_{pd3}

RMS, all trimers	DE	PDE	POEsi	POEdi	POEfu	PRE
Ξ_{omp}	0.63	0.64	0.64	0.89	0.76	11.82
Ξ_{odp}	0.59	0.57	0.69	2.04	0.68	0.16
Best ONIOM scheme	0.17	0.17	0.64	0.89	0.68	0.16
	Ξ_{z}	Ξ_{z}	Ξ_{omp}	Ξ_{omp}	Ξ_{odp}	Ξ_{odp}
Ξ_{pmon}	48.90	56.18	-	-	-	21.87
Ξ_{pdim}	4.99	5.96	-	-	-	21.06
Best monomer scheme	48.60	54.44	-	-	-	20.96
	Ξ_{pm2}	Ξ_{pm2}	-	-	-	Ξ_{pm2}
Best dimer scheme	2.45	1.25	-	-	-	20.65
	Ξ_{pd3}	Ξ_{pd3}	-	-	-	Ξ_{pd3}

Table S23: Errors for basic schemes and best schemes in additive and subractive/ONIOM category for each method for all dispersion dominated trimer systems in kJ/mol.

Ξ_{omp}	0.73	0.51	0.52	0.54
Ξ_{odp}	0.39	0.19	0.37	0.37
Best ONIOM scheme	0.14	0.19	0.18	0.21
	Ξ_{odw}	Ξ_{z}	Ξ_{odww}	Ξ_{odww}
Ξ_{pmon}	-	0.98	6.38	3.61
Ξ_{pdim}	1.62	0.95	1.20	0.99
Best monomer scheme	-	0.98	6.38	3.61
	-	Ξ_{pmon}	Ξ_{pmon}	Ξ_{pmon}
Best dimer scheme	1.62	0.35	0.57	0.44
	Ξ_{pdim}	Ξ_{pd3}	Ξ_{pd3}	Ξ_{pd3}

RMS of $R_{ID} < 0.33$	PE	PPE	DPE	QPE
Ξ_{omp}	0.44	0.52	0.45	0.44
Ξ_{odp}	0.31	0.35	0.33	0.31
Best ONIOM scheme	0.07	0.13	0.09	0.07
	Ξ_{z}	Ξ_{z}	Ξ_{z}	Ξ_{z}
Ξ_{pmon}	21.96	21.02	20.79	21.96
Ξ_{pdim}	2.27	2.29	2.18	2.27
Best monomer scheme	21.21	20.36	20.30	21.21
	Ξ_{pm2}	Ξ_{pm2}	Ξ_{pm2}	Ξ_{pm2}
Best dimer scheme	0.23	0.41	0.76	0.23
	Ξ_{pd3}	Ξ_{pd3}	Ξ_{pd3}	Ξ_{pd3}

RMS of $R_{ID} < 0.33$	DE	PDE	POEsi	POEdi	POEfu	PRE
Ξ_{omp}	0.361	0.35	0.27	1.19	0.84	18.73
Ξ_{odp}	0.34	0.34	0.84	1.19	0.86	9.08
Best ONIOM scheme	0.12	0.12	0.13	0.11	0.10	0.17
	Ξ_{z}	Ξ_{z}	Ξ_{omp3}	Ξ_{omp2}	Ξ_{z}	Ξ_{z}
Ξ_{pmon}	22.82	24.58	-	-	-	23.25
Ξ_{pdim}	1.55	1.92	-	-	-	21.61
Best monomer scheme	22.70	24.08	-	-	-	21.54
	Ξ_{pm2}	Ξ_{pm2}	-	-	-	Ξ_{pm2}
Best dimer scheme	0.82	0.41	-	-	-	19.86
	Ξ_{pd3}	Ξ_{pd3}	-	-	-	Ξ_{pd3}

Table S24: Errors for basic schemes and best schemes in additive and subractive/ONIOM category for each method for all mixed trimer systems in kJ/mol.

RMS of mixed	unembedded	PCE	DCE	QCE
Ξ_{omp}	1.72	0.94	1.05	0.92
Ξ_{odp}	1.06	0.75	0.77	0.78
Best ONIOM scheme	0.60	0.58	0.40	0.70
	Ξ_{odw}	Ξ_{odww}	Ξ_{odww}	Ξ_{odww}
Ξ_{pmon}	-	6.68	9.42	13.71
Ξ_{pdim}	4.81	1.87	1.39	2.46
Best monomer scheme	-	6.68	9.42	13.71
	-	Ξ_{pmon}	Ξ_{pmon}	Ξ_{pmon}
Best dimer scheme	4.81	0.49	0.87	0.73
	Ξ_{pdim}	Ξ_{pd3}	Ξ_{pd3}	Ξ_{pd3}

RMS of mixed	PE	PPE	DPE	QPE
Ξ_{omp}	1.39	1.15	1.06	1.08
Ξ_{odp}	0.75	0.73	0.71	0.69
Best ONIOM scheme	0.16	0.19	0.15	0.11
	Ξ_{z}	Ξ_{z}	Ξ_{z}	Ξ_{z}
Ξ_{pmon}	43.60	58.76	55.21	63.89
Ξ_{pdim}	4.50	7.51	6.31	8.22
Best monomer scheme	43.53	56.52	54.01	60.89
	Ξ_{pm2}	Ξ_{pm2}	Ξ_{pm2}	Ξ_{pm2}
Best dimer scheme	4.36	0.81	2.02	1.05
	Ξ_{pd3}	Ξ_{pd3}	Ξ_{pd3}	Ξ_{pd3}

RMS of mixed	DE	PDE	POEsi	POEdi	POEfu	PRE
Ξ_{omp}	0.80	0.82	0.57	0.63	0.47	9.29
Ξ_{odp}	0.74	0.71	0.45	2.48	0.45	4.68
Best ONIOM	0.20	0.20	0.21	0.63	0.21	4.68
scheme	Ξ_{z}	Ξ_{z}	Ξ_{omp}	Ξ_{z}	Ξ_{odp}	Ξ_{odp}
Ξ_{pmon}	59.21	67.61	-	-	-	16.79
Ξ_{pdim}	6.75	7.54	-	-	-	16.32
Best monomer scheme	58.86	65.57	-	-	-	16.23
	Ξ_{pm2}	Ξ_{pm2}	-	-	-	Ξ_{pm2}
Best dimer scheme	3.12	1.63	-	-	-	16.23
	Ξ_{pd3}	Ξ_{pd3}	-	-	-	Ξ_{pd3}

Table S25: Errors for basic schemes and best schemes in additive and subractive/ONIOM category for each method for all induction dominated trimer systems in kJ/mol.

RMS of $R_{\mathrm{ID}} > 0.66$	unembedded	PCE	DCE	QCE
Ξ_{omp}	3.87	0.46	0.55	0.42
Ξ_{odp}	3.15	0.39	0.40	0.39
Best ONIOM scheme	2.46	0.37	0.20	0.38
	Ξ_{odw}	Ξ_{z}	Ξ_{odww}	Ξ_{z}
Ξ_{pmon}	-	4.48	5.77	11.39
Ξ_{pdim}	6.59	0.79	0.65	1.18
Best monomer scheme	-	4.48	5.77	11.39
	-	Ξ_{pmon}	Ξ_{pmon}	Ξ_{pmon}
Best dimer scheme	6.59	0.38	0.38	0.26
	Ξ_{pdim}	Ξ_{pd3}	Ξ_{pd3}	Ξ_{pd3}

RMS of $R_{\mathrm{ID}} > 0.66$	PE	PPE	DPE	QPE
Ξ_{omp}	0.80	0.55	0.47	0.48
Ξ_{odp}	0.35	0.32	0.32	0.31
Best ONIOM scheme	0.127	0.09	0.06	0.08
	Ξ_{z}	Ξ_{z}	Ξ_{z}	Ξ_{z}
Ξ_{pmon}	31.94	47.05	45.32	53.59
Ξ_{pdim}	3.10	5.32	4.67	6.03
Best monomer scheme	31.85	44.75	43.14	50.38
	Ξ_{pm2}	Ξ_{pm2}	Ξ_{pm2}	Ξ_{pm2}
Best dimer scheme	2.92	0.34	0.69	1.12
	Ξ_{pd3}	Ξ_{pd3}	Ξ_{pd3}	Ξ_{pd3}

RMS of $R_{\mathrm{ID}} > 0.66$	DE	PDE	POEsi	POEdi	POEfu	PRE
Ξ_{omp}	0.34	0.35	1.35	1.45	1.62	12.62
Ξ_{odp}	0.340	0.32	1.40	1.53	1.30	6.60
Best ONIOM scheme	0.12	0.11	1.00	0.76	1.28	0.19
	Ξ_{z}	Ξ_{z}	Ξ_{z}	Ξ_{odww}	Ξ_{omp2}	Ξ_{odww}
Ξ_{pmon}	43.09	52.93	-	-	-	38.50
Ξ_{pdim}	2.80	5.09	-	-	-	37.50
Best monomer scheme	42.75	50.80	-	-	-	37.26
	Ξ_{pm2}	Ξ_{pm2}	-	-	-	Ξ_{pm2}
Best dimer scheme	2.00	0.84	-	-	-	37.50
	Ξ_{pd3}	Ξ_{pd3}	-	-	-	Ξ_{pdim}

S5 Method and code combinations

Table S26: Each Method and Program combination

Method	TURBOMOLE	DALTON	VASP	MOLPPRO
point charge	X	X		X
dipole	X	X		
quadrupole	X	X		
pol.		X		
point charge pol.		X		
dipole pol.		X		
quadrupole pol.		X		
pol. Dens. no pol.		X		
pol. Dens.		X		
pot. Dens.			X	
proj. Dens.				X

S6 Trimer Interaction Energies PBE0 vs. PBE

The interaction energy error of an subtractive scheme depends strongly on the accuracy of the low level calculation as well as the difference between low level and high level of theory. In this work, we choose PBE as low level and PBE0 as high level of theory. The two functionals are very similar in their construction and are therefore well suited for embedding methods like the potential based embedding since electron densities obtained with PBE will be similar to PBE0 ones. To give an overview of the energies evolved we provide here a table for each group, giving an average deviation of 1.3 kJ/mol.

Table S27: Each group and an example for trimer interaction level of theory differences, PBE is the "low" level of theory and "PBE0" is the high level of theory.

Group and trimer	Ξ^{low} kJ/mol	Ξ^{high} kJ/mol	ΔE kJ/mol	$\Delta\Xi$ in $\%\Xi^{low}$
1a	-48.28	-48.66	-0.39	0.8
1b	-26.08	-25.97	0.11	0.4
1c	-67.27	-67.47	-0.20	0.3
3a	-45.36	-45.76	-0.41	0.9
3b	-9.94	-9.04	0.90	9.1
3c	-19.58	-19.38	0.20	1.0
6a	-43.55	-45.10	-1.56	3.6
6b	-85.25	-88.40	-3.15	3.7
6c	-45.38	-47.22	-1.85	4.1
7a	-7.71	-8.76	-1.05	13.6
7b	-69.26	-71.05	-1.79	2.6
7c	-66.04	-67.70	-1.66	2.5
9a	-64.51	-67.29	-2.78	4.3
9b	-53.83	-56.14	-2.31	4.3
9c	-59.64	-62.25	-2.61	4.4
10a	-49.69	-51.12	-1.43	2.9
10b	-7.58	-7.30	0.28	-3.7
10c	-87.12	-88.34	-1.22	1.4
12a	-67.78	-67.63	0.15	-0.2
12b	0.59	0.30	-0.29	-48.6
12c	-40.65	-41.39	-0.74	1.8
14a	-21.97	-22.83	-0.85	3.9
14b	-75.43	-78.57	-3.14	4.2
14c	-80.62	-84.67	-4.05	5.0
20a	-52.31	-49.64	2.67	-5.1
20b	28.56	28.88	0.32	1.1
20c	28.56	28.88	0.32	1.1
NH_3	-42.92	-41.83	1,09	-2.5
H_2O	-54.82	-56.14	-1.32	2.4

S7 Charges for point charge embedding

Of course, the accuracy of the point charge embedding and all subsequent methods using this embedding type are dependent on the choice of calculating the point charges by itself. In this section, we provide a table for each group and show the differences in charge as well as

the difference in trimer interaction energy when going from Mulliken to natural population analysis (NPA) charges for an additive scheme using dimers (Ξ_{d}).

Table S28: Each group and an example for trimer interaction energy change due to change in charge. The charges are from Mulliken analysis or NPA. A negative $\Delta\Xi_{\mathrm{pdim}}$ corresponds to a drop in accuracy when switching from Mulliken to NPA.

Group and trimer	$\Delta\ \Xi_{\mathrm{pdim}}$ in $\%\Xi_{\mathrm{pdim}}$
Induction 1a	-4.31
Indcution 1b	-6.26
Mixed 1c	-2.79
Mixed 3a	-5.04
Mixed 3b	-2.57
Mixed 3c	-6.22
Mixed 6a	-3.16
Induction 6b	-3.16
Mixed 6c	-7.64
Dispersion 7a	4.45
Mixed 10a	-2.60

References

(1) Dolgonos, G. A.; Hoja, J.; Boese, A. D. Revised values for the X23 benchmark set of molecular crystals. *Phys. Chem. Chem. Phys.* **2019**, *21*, 24333–24344.

(2) Rezac, J.; Huang, Y.; Hobza, P.; Beran, G. J. O. Benchmark Calculations of Three-Body Intermolecular Interactions and the Performance of Low-Cost Electronic Structure Methods. *J. Chem. Theory Comput.* **2015**, *11*, 3065–3079.
